# ICARUS: flexible protein structural alignment based on Protein Units

**DOI:** 10.1093/bioinformatics/btad459

**Published:** 2023-07-27

**Authors:** Gabriel Cretin, Charlotte Périn, Nicolas Zimmermann, Tatiana Galochkina, Jean-Christophe Gelly

**Affiliations:** Université Paris Cité and Université des Antilles and Université de la Réunion, INSERM, BIGR, F-75015 Paris, France; Laboratoire d’Excellence GR-Ex, 75015 Paris, France; Université Paris Cité and Université des Antilles and Université de la Réunion, INSERM, BIGR, F-75015 Paris, France; Laboratoire d’Excellence GR-Ex, 75015 Paris, France; TBI, Université de Toulouse, CNRS, INRAE, INSA, 31077 Toulouse, France; Université Paris Cité and Université des Antilles and Université de la Réunion, INSERM, BIGR, F-75015 Paris, France; Laboratoire d’Excellence GR-Ex, 75015 Paris, France; Université Paris Cité and Université des Antilles and Université de la Réunion, INSERM, BIGR, F-75015 Paris, France; Laboratoire d’Excellence GR-Ex, 75015 Paris, France; Université Paris Cité and Université des Antilles and Université de la Réunion, INSERM, BIGR, F-75015 Paris, France; Laboratoire d’Excellence GR-Ex, 75015 Paris, France

## Abstract

**Motivation:**

Alignment of protein structures is a major problem in structural biology. The first approach commonly used is to consider proteins as rigid bodies. However, alignment of protein structures can be very complex due to conformational variability, or complex evolutionary relationships between proteins such as insertions, circular permutations or repetitions. In such cases, introducing flexibility becomes useful for two reasons: (i) it can help compare two protein chains which adopted two different conformational states, such as due to proteins/ligands interaction or post-translational modifications, and (ii) it aids in the identification of conserved regions in proteins that may have distant evolutionary relationships.

**Results:**

We propose ICARUS, a new approach for flexible structural alignment based on identification of Protein Units, evolutionarily preserved structural descriptors of intermediate size, between secondary structures and domains. ICARUS significantly outperforms reference methods on a dataset of very difficult structural alignments.

**Availability and implementation:**

Code is freely available online at https://github.com/DSIMB/ICARUS.

## 1 Introduction

Protein structure is crucial for understanding evolution and function mechanisms at a molecular level. It is estimated that the number of protein folds is limited (Magner *et al.* 2015) and distributions of folds are highly skewed (Wolf *et al.* 2000, Coulson and Moult 2002, Koonin *et al.* 2002, Leonov *et al.* 2003). In addition, as “Structure is three to ten times more conserved than sequence” (Illergård *et al.* 2009), the evolutionary link between far remote homolog proteins can only be highlighted by observing their structure. Comparison of two protein structures using structural alignment is therefore important to deduce information that sequence alignment alone cannot provide. Indeed, superposition of two protein structures enables characterization of their relationships at an atomic level in order to highlight similarities to potentially infer their function, as well as to unveil complex evolutionary links. Structural alignment of two distantly related proteins (homologs) allows to reveal mutation tolerance as well as to identify flexible regions, together with the positions of conserved residues important for their function or architecture. Besides, there are many proteins evolutionarily unrelated but sharing similar fold and function, called analogs (Russell *et al.* 1997). The structural alignment of two analogs can unveil the protein sequence space of a fold, but also reveal the positions of residues that are fundamental for it (Sierk and Kleywegt 2004).

Structure alignment method is determined by a scoring function used for protein structural similarity measurement and by an algorithm to perform the superposition. The main metrics used to quantify the difference between two protein structures are the Root Mean Square Deviation (RMSD) and the Template Modeling score (TM-score) (Mayr *et al.* 2007, Xu and Zhang 2010). As compared to RMSD which measures the mean squared distance between the C-alpha atoms of superimposed proteins, TM-score is (i) independent from protein size as it implements a length-dependent scale to normalize the distance errors and (ii) less sensitive to local deviation as smaller distance errors are weighted more heavily than larger distance errors, making the score value more suitable for global fold similarity measurement than local structural variations. Therefore, TM-score became the reference to quantify the similarity between two superimposed structures and is currently used by most methods.

Most popular tools used for protein structure alignment such as TM-align (Zhang and Skolnick 2005), DALI (Holm 2020), and Combinatorial Extension (CE) (Shindyalov and Bourne 1998) are based on rigid sequential alignment, which consists in finding an optimal rotation and translation of one structure to minimize its distance to another structure. These methods mostly rely on least-squares fitting algorithms or contact map overlap such as PAUL (Wohlers *et al.* 2010) and CSA (Wohlers *et al.* 2010, 2012) and considers both structures as rigid bodies. Since such algorithms consider exclusively global 3D geometric similarity, they fail to correctly superimpose protein structures related by complex evolutionary events. Indeed, despite global structural similarity, homologous proteins can show very high local structural variability in case of repeats of the same structural subunit, circular permutations or insertion of large protein fragments (Grishin 2001). In addition, rigid alignment algorithms demonstrate poor performance for superposition of highly flexible proteins able to adopt a variety of diverse conformations.

In order to highlight protein structural similarity in case of structural flexibility and/or complex evolutionary relationship, a number of flexible structural alignment methods were developed such as DEDAL (Daniluk and Lesyng 2011), KPAX (Ritchie 2016), and FATCAT (Ye and Godzik 2003, Li *et al.* 2020). These flexible methods rely on the detection of “hinge” positions (hinges) around which rigid regions of the structure to be aligned orient themselves relative to each other in order to achieve the best possible overall alignment. The quality of a flexible structural alignment highly depends on the correct identification of these protein regions which should remain rigid during structural superposition.

However, some protein cases remain difficult to align correctly. Indeed, even recent flexible methods are still unable to produce relevant structural alignment, especially in the cases of proteins with intrinsically disordered regions or with nonsequential relationships such as circular permutation. As a particularly interesting example, there are haloacid dehalogenase and chemotaxis protein CheY which share the same Mg2+-binding site and conserved residues involved in phosphate binding (Ridder and Dijkstra 1999) which neither flexible method is able to identify.

In this study, we present ICARUS (flexIble struCtural Alignment based on pRotein UnitS) a flexible alignment method which uses the Protein Peeling algorithm (Gelly *et al.* 2006a,b, Gelly and de Brevern 2011, Postic *et al.* 2017, Cretin *et al.* 2022) to identify compact regions of a protein structure called Protein Units (PUs). Protein Units define independent rigid regions to be aligned to the target.

Our strategy relies on the pretreatment of one of the protein structures to align by splitting it into relevant compact elements which are less prone to be flexible and constitute rigid and stable structural units (Gelly *et al.* 2006a,b, Gelly and de Brevern 2011, Postic *et al.* 2017). They were successfully used to handle ambiguous protein structure partitioning (Cretin *et al.* 2022).

In addition, PUs tend to be less split into nonsequential alignments in evolutionary events. Indeed, as shown in Gelly *et al.* (2012), PUs are preserved during alternative splicing events. Thus, Protein Peeling is a relevant way to identify rigid regions (a.k.a. PUs) to align instead of proceeding with classical flexible alignment strategies which try to identify pivot protein fragments.

Our method significantly outperforms both rigid and flexible reference methods such as DEDAL, FATCAT, and KPAX for the most complex cases of protein structural comparisons considering performances in terms of TM-score as well as on the available corresponding reference alignments.

## 2 Materials and methods

### 2.1 ICARUS algorithm

**Figure 1.**
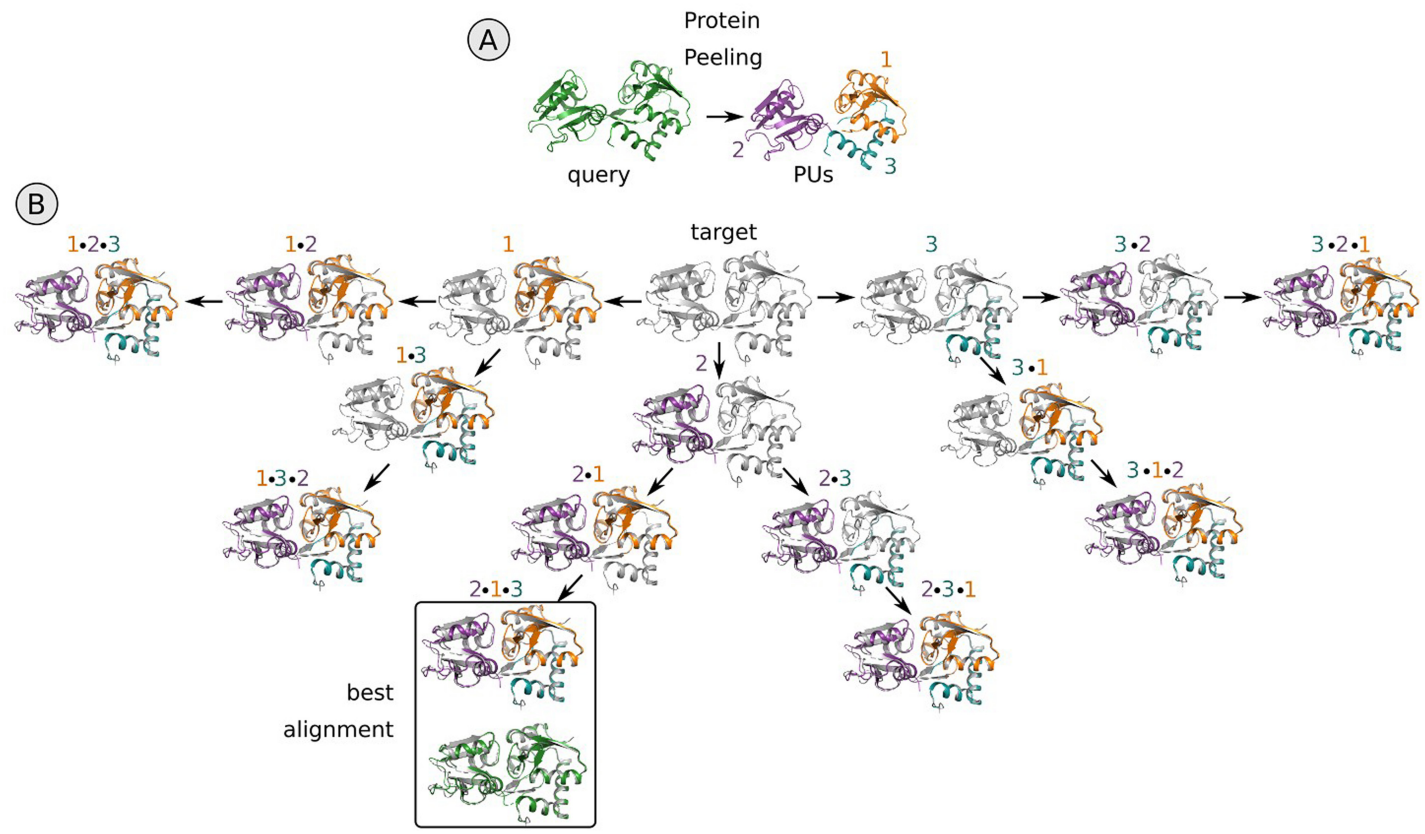
Main ICARUS algorithm applied to the alignment of the query protein d1ggg (in green, left in part A) on the target d1wdn (in gray, middle of part B). (A) Protein Peeling algorithm identifies three PUs (given by colored numbers 1, 2, and 3) composing the structure of the query protein (d1ggg). (B) ICARUS performs the subsequent alignments of the identified PUs one by one exploring all the possible strategies. At each stage of the process, the next PU is aligned to the part of the protein which was not yet aligned to any previous aligned PU (gray part of the target protein). The procedure is then repeated switching target and query proteins. Once all the possible strategies are explored, the best alignment is chosen according to the best TM-score.

The main ICARUS algorithm builds a number of structural alignments between two proteins which are both considered alternatively as query and target, using Protein Peeling for the rigid regions’ identification. Protein Peeling method works solely from the contact probability matrix, i.e. the Cα distances translated into probabilities using a logistic function. It uses hierarchical top-down divisive clustering to create a series of nested partitions of the 3D structure. Every step aims at dividing a unit optimally into 2 or 3 sub-units according to a criterion called “partition index” assessing the structural independence of the sub-units newly defined. An illustration of Protein Peeling top-down clustering on the proteins d1b5ta_ and d1k87a2 from the RIPC dataset is shown in Supplementary Fig. S10. A statistical criterion assesses the protein structure dissection. First, using the second input protein as a rigid “target,” Protein Peeling is applied to the first input protein, the “query,” in order to subdivide it into Protein Units (PUs): compact fragments with high density of internal contacts and low number of contacts between each other. Protein Peeling returns protein segmentation at several hierarchical levels containing an increasing number of PUs of decreasing sizes. Then, ICARUS performs subsequent alignments of the identified PUs to the target iteratively using KPAX (in rigid mode). At each stage of the process, the next PU is aligned to the portion of the protein which has not yet been associated with any previously aligned PU. We explore all the possible alignment strategies that can be obtained by changing the order of PU alignments (Fig. 1), while skipping alignments already done previously using a branch and bound algorithm. The pseudo-code of ICARUS main algorithm is available in Supplementary Fig. S8.

In order to select the tradeoff between runtime and quality of the solution, we determined four different exploration levels {1,2,3,4} which the user can choose from. The level of exploration determines how many PUs are considered in the graph exploration according to Protein Peeling’s segmentation output. Indeed, Protein Peeling computes a progressive top-down splitting of the protein which produces PUs of decreasing sizes. With increasing exploration level, the number of PUs considered by ICARUS increases and average PU size decreases. Level 1 allows 2 or 3 PUs, level 2 allows 4 or 5 PUs, 6 PUs for level 3, and 7 PUs for level 4.

Therefore, at high exploration levels ICARUS explores more solutions and potentially finds better alignments. However, the highest exploration level also increases exponentially the program runtime (Supplementary Fig. S9). A low exploration level can on the contrary potentially miss optimal alignments.

In order to determine the optimal exploration level among L{1,2,3,4}, we calculated the Akaike Information Criterion (AIC) and the corrected AIC for small samples (AICc) values for each exploration level (Supplementary Fig. S4) in terms of performances on the 23 RIPC protein pairs for which a reference alignment is provided. The AIC and AICc values of L2 are much lower than the other levels for a lower number of parameters (4–5 PUs instead of >6 PUs) so the best model is ICARUS Level 2.

The elbow curves (Supplementary Fig. S5) corroborate this result. Hence, the default exploration level was set to 2, providing users the optimal compromise between performance and runtime.

Finally, ICARUS repeats the procedure after switching target and query proteins. This operation is necessary to perform better. On average, we obtain a difference of TM-score of 0.048, 0.067, 0.267, and 0.238 for levels 1, 2, 3, and 4, respectively when proteins to align are swapped (Supplementary Table S16). Yet, there are several examples for which the difference is much higher, e.g. the pair d1nkl__ and d1qdma1, at level 2 the difference is of 0.777 which allows ICARUS to find 91.3% of reference residues, while at level 1 none was found. In this case, swapping proteins to align was crucial to find reference alignment residues.

Once all the alignments for all PU exploration levels are calculated, the best alignment is chosen on the basis of the TM-score (Xu and Zhang 2010) optimized by a house-made script and normalized by the length of the shortest protein.

ICARUS was implemented in Python 3 with parts of the algorithm written in C++ and Perl. The code is freely available online at https://github.com/DSIMB/ICARUS. The PUs were determined using Protein Peeling version 3. The typical running time for a single comparison of a pair of protein structures ranges from a few seconds for exploration levels 1–3, to a few minutes for level 4, depending on the length of proteins and their topology (Supplementary Fig. S9).

### 2.2 Evaluation of the results

The proposed strategy has been tested on two different datasets. First, the RIPC dataset (Mayr *et al.* 2007) identifies 40 pairs of similar but complex to align SCOP (Structural Classification of Proteins database) (Fox *et al.* 2014, Chandonia *et al.* 2022) domains due to very difficult structural relations. The cases identified in this dataset are protein pairs that diverge due to Repetitions, very large Insertions, Permutations, and extensive Conformational Variability (RIPC). Then, we tested the methods on the SISY-pairwise dataset, which derives from the SISYPHUS database (Andreeva *et al.* 2007), structural alignments for proteins with nontrivial relationships.

Our results were compared to the classical alignment tool TM-align, to the reference flexible alignment method FATCAT (Ye and Godzik 2003, Li *et al.* 2020, Mayr *et al.* 2007) as well as to DEDAL (Daniluk and Lesyng 2011), and the well performing tool KPAX (Ritchie 2016). We launched all tools with their default parameters or parameters used in publication paper. The versions of the programs used are as follows: FATCAT Github version of Mar 9, 2022, TM-align version 20190822, DEDAL version 1.0.5. We retrieved PAUL performances on the RIPC dataset directly from the paper.

We used TM-score normalized by the shortest sequence length to evaluate the similarity between two aligned structures, as well as the percentage of residues matching the aligned residues in the reference alignments provided by both datasets. TM-score adopts values between 0 and 1, where 1 means that two structures are identical. Two structures are considered to share the same fold if their alignment TM-score ≥ 0.5 and beyond this threshold value (Xu and Zhang 2010). The RIPC dataset reports reference alignments for only 23 out of the 40 pairs in total. Out of the 130 reference alignments provided by the SISY dataset, we excluded 19 pairs composed of at least one multi-chain protein as ICARUS does not treat multi-chains, which leaves 111 reference alignments.

## 3 Results

### 3.1 RIPC dataset

**Figure 2.**
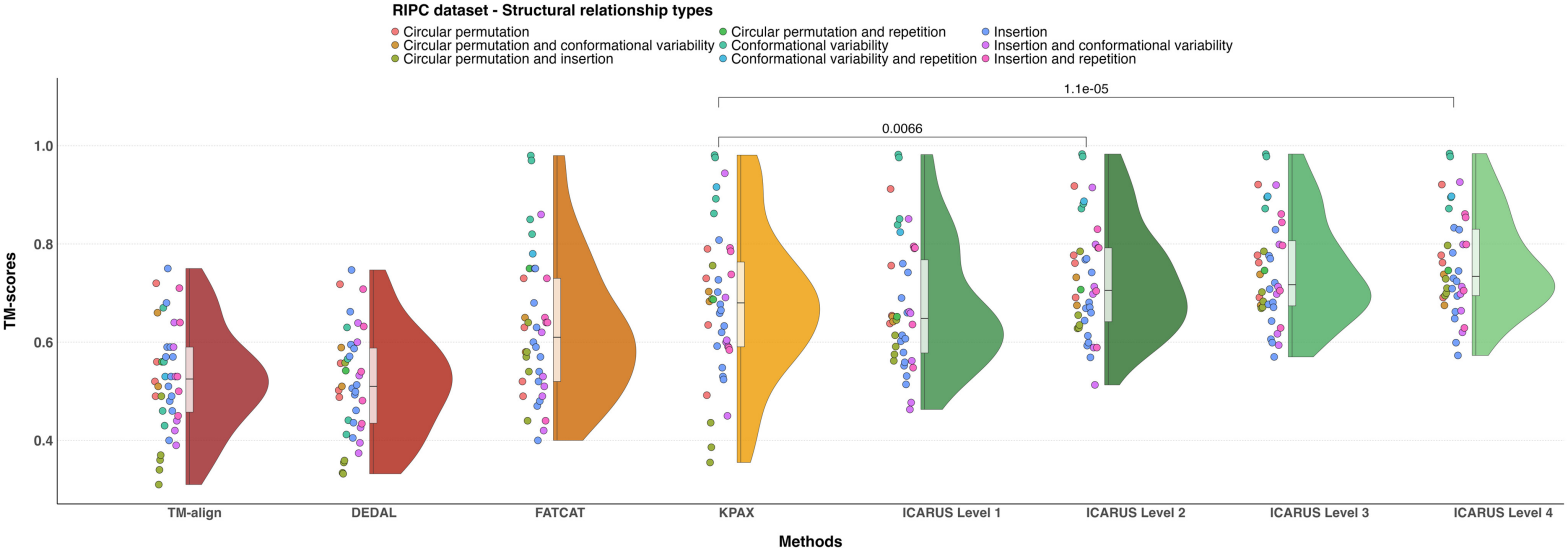
TM-scores of flexible structural alignments obtained by the four levels of ICARUS compared to that of state-of-the-art methods TM-align, DEDAL, FATCAT, and KPAX on 40 protein pairs of the RIPC dataset. The *P*-values of two Wilcoxon paired signed-rank tests are shown on top between KPAX and exploration levels 2 and 4. The aligned protein pairs are colored according to the class of difficulty they belong to, a combination of R, I, P, and C.

**Figure 3.**
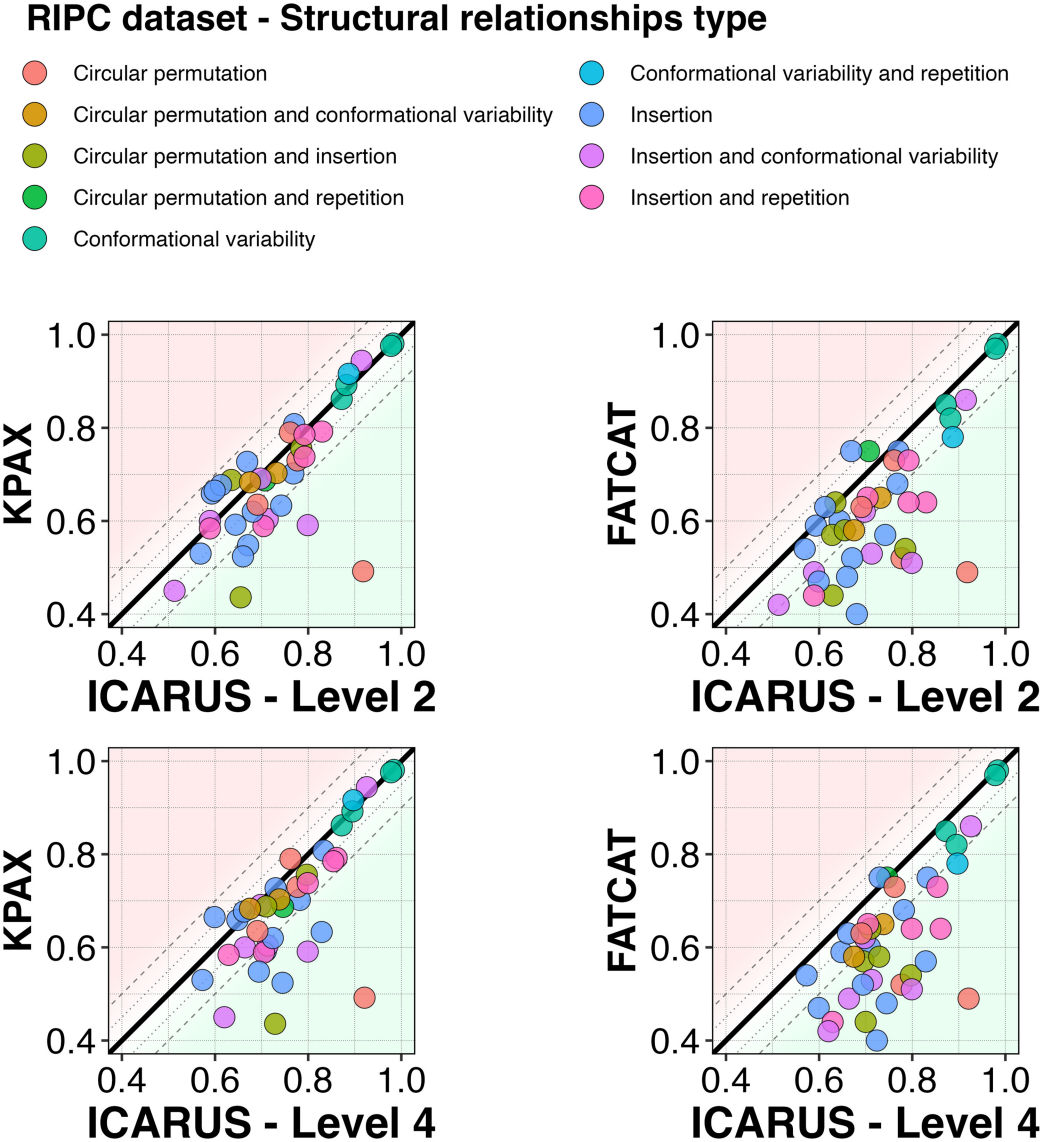
TM-scores of flexible structural alignments obtained by KPAX and FATCAT against those obtained by ICARUS using the default exploration level 2 (4–5 PUs) and 4 on the 40 particularly difficult protein pairs of the RIPC dataset. The diagonal represents equal scores. The dashed lines show differences of 0.05 and 0.1 between the scores obtained by the two methods. The aligned protein pairs are colored according to the class of difficulty they belong to, a combination of R, I, P, and C.

For a given exploration level *n*, ICARUS outputs the best alignment(s) obtained for levels {1, …, *n*}. At level 1, ICARUS already outperformed all methods significantly (except KPAX) on the full RIPC dataset (Fig. 2 and Supplementary Fig. S1) with an average TM-score of 0.76 against 0.63, 0.52, 0.68, and 0.53 for FATCAT, DEDAL, KPAX, and TMalign, respectively (Table 1). Increasing the number of PUs used to build the alignments increases precision further (Fig. 2 and Supplementary Fig. S2). Indeed, average ICARUS TM-scores are 0.675, 0.728, 0.747, and 0.759 for exploration levels 1, 2, 3, and 4, respectively (Supplementary Tables S1–S10). From level 2, ICARUS outperforms reference methods in 28/40 cases for reasonable execution times (Fig. 3 and Supplementary Fig. S9). We thus recommend to use ICARUS exploration level 4 only in order to fine-tune best alignments or for especially difficult cases, otherwise use exploration level 2 for systematic analysis.

**Table 1.** TM-scores of flexible structural alignments obtained by TM-align, FATCAT, DEDAL, and KPAX against those obtained by ICARUS level 4 on the 40 difficult protein pairs of the RIPC dataset.^a^

Structural relation types	Nb. pairs	ICARUS	TM-align	FATCAT	DEDAL	KPAX
Conformational variability	4	**0.93**	0.53	0.91	0.51	**0.93**
Circular permutation	4	**0.79**	0.57	0.59	0.57	0.66
Circular permutation and insertion	5	**0.73**	0.37	0.55	0.39	0.52
Insertion	12	**0.71**	0.55	0.58	0.54	0.64
Insertion and conformational variability	6	**0.74**	0.50	0.57	0.49	0.65
Insertion and repetition	5	**0.77**	0.57	0.62	0.56	0.70
Circular permutation and conformational variability	2	**0.71**	0.59	0.62	0.55	0.69
Circular permutation and repetition	1	**0.75**	0.56	**0.75**	0.54	0.69
Conformational variability and repetition	1	0.90	0.53	0.78	NA	**0.92**
**Weighted average**		**0.76**	0.53	0.63	0.52	0.68

a DEDAL is unable to produce an alignment for the “Conformational variability and repetition” protein pair (d1aj3__ and d2spca_). The average value is ponderated by the number of pairs in each category.

Best values are in bold.

The alignment scores obtained by ICARUS exploration levels 2 and 4 ranged from 0.513 to 0.983 with an average of 0.748 and from 0.573 to 0.984 with an average of 0.779, respectively, while the scores of the next two best methods start with much lower scores: FATCAT ranged from 0.40 to 0.98 with an average of 0.69 and those of KPAX ranged from 0.36 to 0.98 with an average of 0.67 (Supplementary Tables S1–S9). The differences of TM-score averages between ICARUS and all other methods are highly statistically significant (Supplementary Fig. S1, *P*-values lower than *α* = 0.05 on paired signed-rank T-test). Importantly, ICARUS is the only tool able to detect a structural relation at fold level for the particularly complex cases of the circular permutations associated with insertions (Supplementary Table S3) as their alignment score never fell below 0.5.

**Figure 4.**
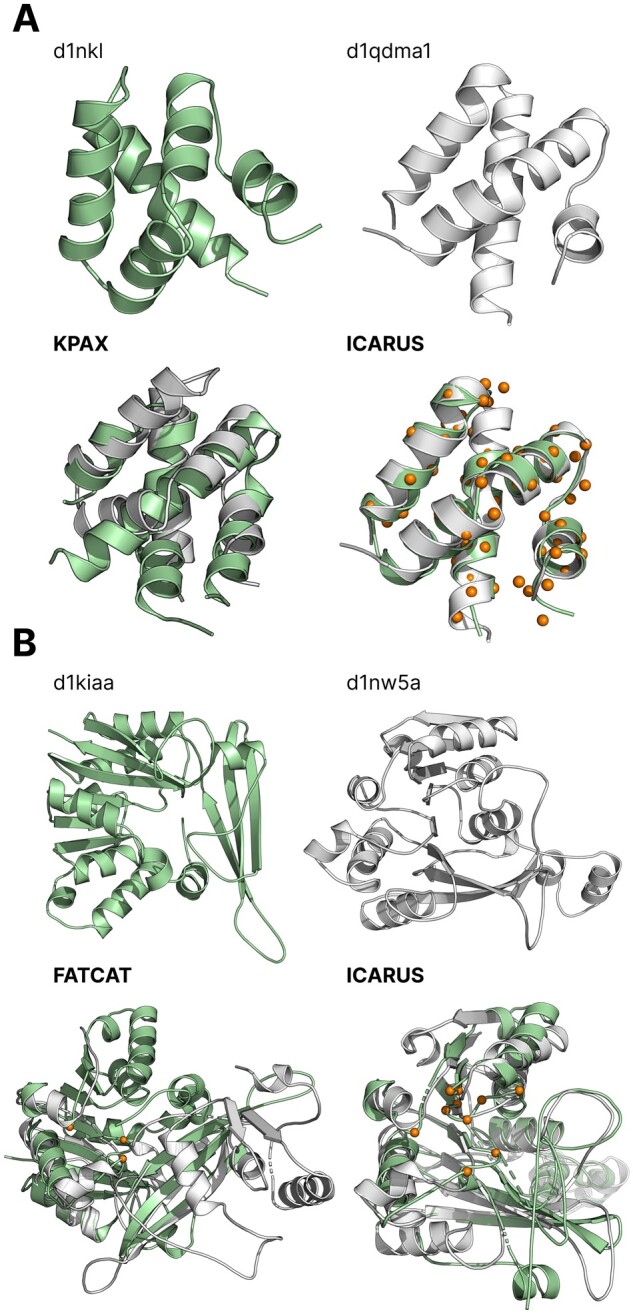
Two examples of ICARUS, FATCAT, and KPAX alignments of SCOP domains from the RIPC dataset. (A) Alignment of two domains related by a circular permutation: d1nkl__ (green) aligned to d1qdma1 (white) by ICARUS and KPAX. (B) Alignment of two domains related by a circular permutation and insertion: d1kiaa_ (green) aligned to d1nw5a_ (white) by ICARUS and KPAX. In both (A) and (B) sticks and spheres represent the reference alignment residues that were correctly aligned by tools. In (A) ICARUS finds 93.1% of them (67/72), KPAX none. In (B) ICARUS correctly aligns 83% of reference alignment residues (10/12) while FATCAT finds only 25% (3/12).

Figure 4 perfectly illustrates ICARUS high performance on very complex alignments, even between small protein domains. For example, the alignment of the 77 and 78 residue long domains nk-lysin (d1nkl__) and prophytepsin (d1qdma1) respectively (Fig. 4A) requires to operate a large circular permutation in order to correctly align the 72 reference residues. The structure of d1nkl__ should be split into two halves which then need to be swapped in order to be aligned correctly to the d1qdma1 structure. ICARUS was the only method able to perform such an operation and correctly aligned 93.1% (67/72) of reference residues with a TM-score of 0.757 as illustrated in the ICARUS terminal output example presented in Supplementary Fig. S6. Splitting d1nkl__ into four PUs enabled the detection of the very large circular permutation. KPAX on the other hand, completely misses the permutation and misaligns all reference residues. DEDAL finds only 48.6% of reference residues.

Another even more complex example is presented in Fig. 4B with the alignment between d1kiaa_, a domain of Glycine N-methyltransferase and d1nw5a_, a beta class N6-adenine DNA methyltransferase, related by a circular permutation and an insertion. The reference alignment proposed by Mayr *et al.* contains only 12 residues corresponding to the binding sites for N-methyltransferase activity, that should be correctly aligned among the 275 (d1kiaa_) and 270 (d1nw5a_) residue long proteins. ICARUS was able to match 83.3% of reference alignment residues (10/12) while FATCAT, KPAX, and TMalign only 25% (3/12) and DEDAL only 58.3% (7/12). The sequence alignment produced by ICARUS can be found in Supplementary Fig. S7. Such examples illustrate how a performant flexible protein alignment method can impact the quality and relevance of a protein functional annotation. On average, ICARUS has a better agreement with the reference alignments on the very hard protein pairs of the RIPC dataset than concurrent methods (Supplementary Table S10). On average, ICARUS aligns 73.6% of the reference positions accurately, whereas KPAX only 69.4% of them, FATCAT 56.6%, DEDAL 67.2%, and TM-align 57.7%. Although the mean differences are not all significant (Supplementary Table S10), we show previously that performance in terms of both TM-scores and reference positions agreement has a potential major impact on the functional and evolutionary annotation of alignments. A nonsequential approach such as ICARUS enables such operations.

### 3.2 SISY dataset

We also tested our method on the SISY dataset (Supplementary Fig. S15). This dataset is composed of protein pairs with nontrivial relationships derived from the SISYPHUS dataset. Interestingly, for this dataset, splitting protein structures into increasingly smaller PUs was detrimental to both TM-scores and the percentage of agreement on the reference alignments provided. The best performing ICARUS exploration level is the first one, which splits protein into a maximum of 2 or 3 PUs. This can be explained by the fact that the less complicated relationships relating the protein pairs of SISY require less swapping and permutations of protein fragments to find best alignment. Also, unlike RIPC which is composed of SCOP domains (average length of 220 residues), SISY dataset is composed of full length proteins (average length of 256 residues), so over-splitting proteins increases the chance to miss the correct alignment. ICARUS (level 4) obtains statistically significantly higher performances in terms of TM-scores compared to all other methods with an average TM-score of 0.758 (Supplementary Tables S14 and S15). The second best mean TM-score of 0.71 is obtained by KPAX. ICARUS also performs well on the reference alignments provided by SISY with an average percentage of agreement of 74.1% for level 1, which is higher than the reference method FATCAT and TM-align with 71.6% and 66.3%, respectively. The method which aligns correctly the most reference residues is DEDAL with a percentage of 77.2% followed by KPAX with 75.8% of positions, although the differences with ICARUS are not significant according to Wilcoxon paired signed-rank tests (Supplementary Table S13).

## 4 Conclusion

Alignment of protein structures is very complex in case of their great conformational variability, or complex evolutionary events such as large insertions, circular permutations or repetitions. For these complex cases, classical methods are not efficient and correct structure superposition is only possible using flexible alignment approaches. The ICARUS method presented here is based on protein structure division into structurally and evolutionarily relevant Protein Units to determine the rigid regions reoriented during alignment procedure.

Our method demonstrates excellent performances on the RIPC dataset with both significantly higher TM-scores obtained for 38/40 of resulting alignments as compared to the FATCAT reference method and 33/40 compared to KPAX, as well as higher percentage of correctly aligned positions according to reference alignments which highlights residues involved in functional and evolutionarily important positions (binding sites, active sites, etc.), thus essential for protein structures annotation. ICARUS also shows great performances on full length proteins from the SISY dataset both in terms of TM-scores and percentage of agreement with the reference alignments.

The ability of ICARUS to detect particularly complex relations between protein pairs comes from its nonsequential algorithm. Indeed, considering query proteins as consecutive protein fragments which can be swapped independently, ensures a more flexible representation of the structure. The only other method that uses a nonsequential algorithm is KPAX which demonstrates second best performances.

The reason why ICARUS performs better on particularly complex cases of circular permutation, insertions, and conformational variability, comes from the use of Protein Units for identification of the rigid regions and hinge positions in the protein structure. PUs were previously shown to constitute rigid and stable structural units (Gelly *et al.* 2006a,b, Gelly and de Brevern 2011, Postic *et al.* 2017) successfully used to handle ambiguous protein structure partitioning (Cretin *et al.* 2022). In the current study PUs allow us to obtain high quality structural alignments for the proteins related by the complex evolutionary events corresponding to reorganizations/repetitions/growths of structural modules of intermediate size. Therefore, we can expect PU to play a role of such evolutionary modules, in the same way as secondary structures and domains do.

## Supplementary Material

btad459_Supplementary_Data

## Data Availability

The ICARUS code and data underlying this article are freely available on Github at https://github.com/DSIMB/ICARUS.
