# Supplementary material for: ICARUS: flexible protein structural alignment based on Protein Units
Source: Bioinformatics. 2023 Jul 27;39(8):btad459. doi: 10.1093/bioinformatics/btad459 (PMC10400377; doi:10.1093/bioinformatics/btad459)
Supplement: btad459_Supplementary_Data [file btad459_supplementary_data.pdf]

SUPPLEMENTARY

	KPAX	FATCAT	DEDAL	TMalign
**ICARUS L1**	8.66E-1	**2.19E-3**	**1.82E-12**	**2.71E-08**
**ICARUS L2**	**3.31E-3**	**2.96E-07**	**1.82E-12**	**9.09E-13**
**ICARUS L3**	**7.72E-05**	**6.37E-11**	**2.73E-08**	**1.85E-08**
**ICARUS L4**	**5.36E-06**	**2.81E-08**	**1.82E-12**	**1.85E-08**

Supplementary Fig. S1. P-values of paired signed-rank Wilcoxon tests executed on TM-scores of each ICARUS exploration level against all concurrent methods on the RIPC dataset. Null hypothesis is that means of ICARUS levels are equal to other methods. Alternative hypothesis is that ICARUS levels TM-scores means are greater than other methods. In bold P-values lower than the significative level of 0.05.

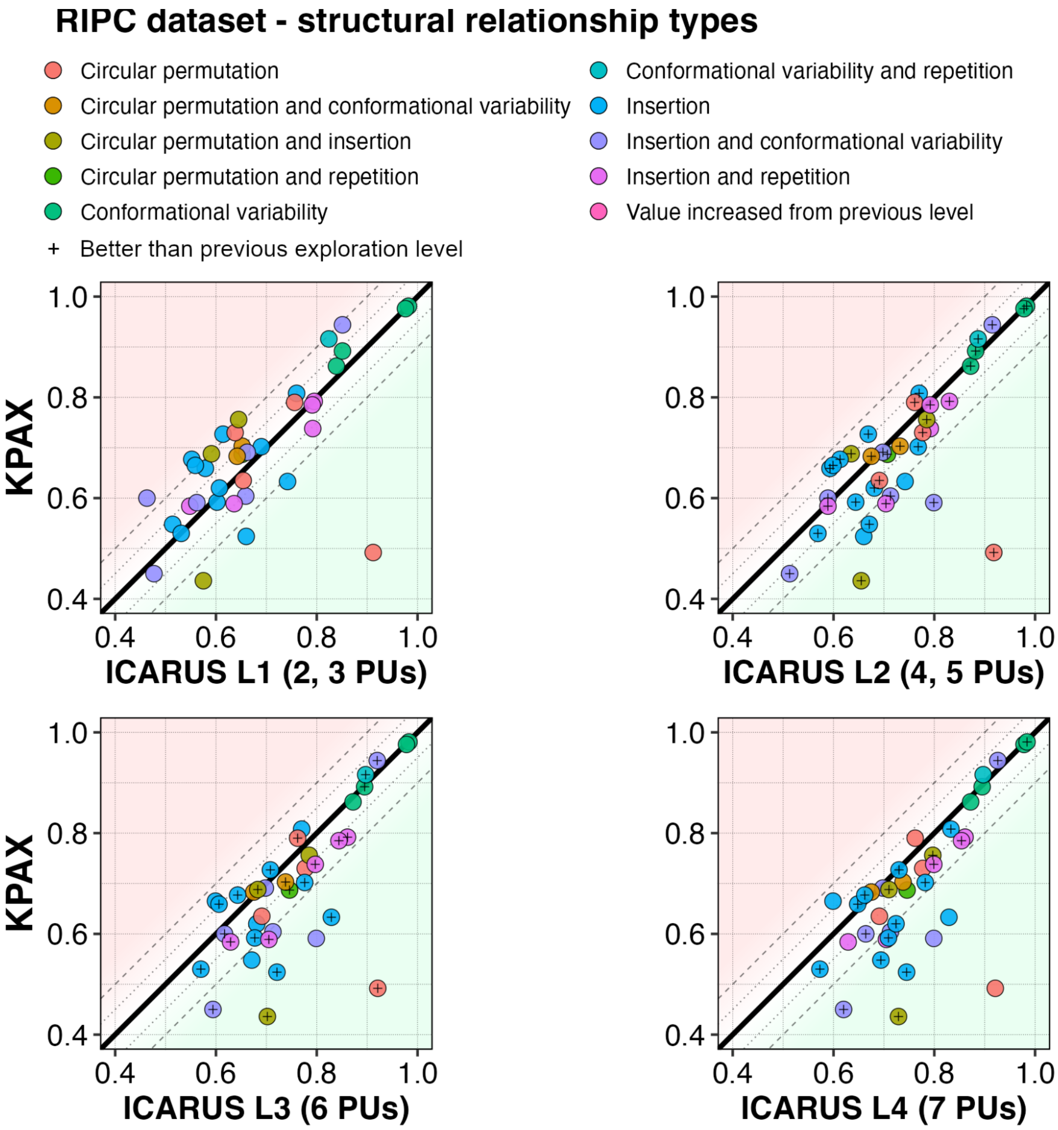

Supplementary Fig. S2. TM-scores of structural alignments obtained by ICARUS against those obtained by KPAX for all exploration levels of ICARUS. Points which contain a "+" sign represent TM-scores whose values have changed compared to the previous exploration level. The diagonal represents equal scores. The dashed lines show differences of 0.05 and 0.1 between the scores obtained by the two methods. The aligned protein pairs are colored according to the class of difficulty they belong to, a combination of R, I, P and C.

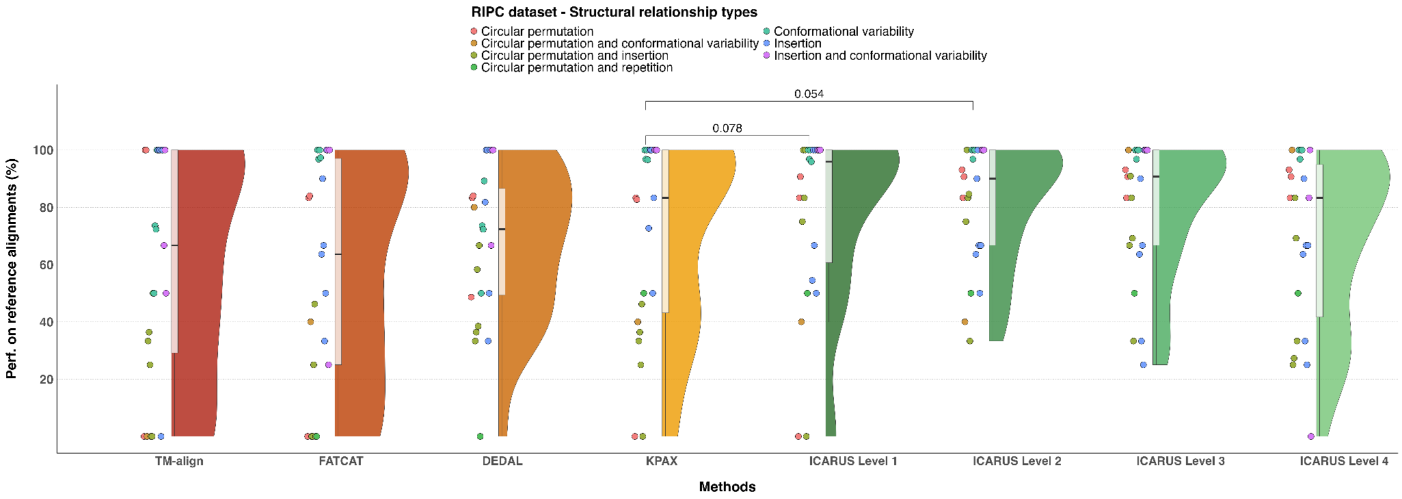

Supplementary Fig. S3. Performance of each method on the 23 reference alignments provided by the RIPC dataset. For each protein pair alignment, the percentage of residues matching the corresponding reference alignment is calculated. There are no reference alignments for protein pairs of type "Insertion and repetition" and "Conformational variability and repetition". The p-values of two Wilcoxon paired signed-rank tests (alternative hypothesis "greater") are shown on top between KPAX and exploration levels 1 and 2.

	ICARUS			
	Level 1	Level 2	Level 3	Level 3
AIC	-88.62	**-114.99**	-102.82	-81.73
AICc	-87.36	**-111.46**	-97.57	-74.27

Supplementary Fig. S4. Akaike Information Criterion (AIC) and AIC corrected for small samples (AICc) calculated on performances of each level of ICARUS for the 23 RIPC protein pairs which have a reference alignment. In bold the lowest values representing the best model.

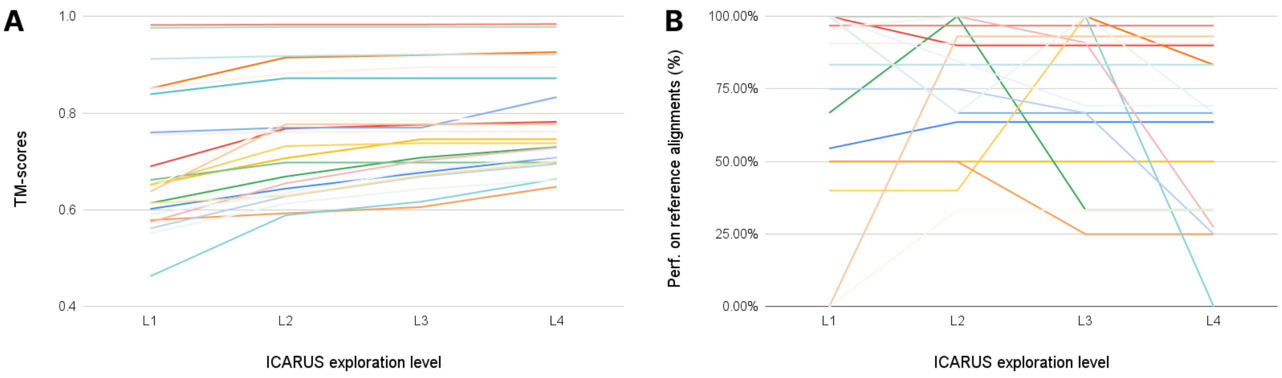

Supplementary Fig. S5. A. Evolution of TM-scores obtained by each exploration level of ICARUS on the 23 RIPC protein pairs which have a reference alignment. **B.** Evolution of the performance of ICARUS exploration levels on the 23 reference alignments of RIPC. The most important increase in TM-scores and performance on reference alignment values happen between L1 and L2 represented by an elbow with the curves.

```
  * Score: 0.777
    Query: d1nkl__
     ├── PU order:     PU1 |      PU2 |      PU3 |      PU4 |
     └── Regions :    1-17 |    18-46 |    47-64 |    65-78 |
    Target: d1qdma1
     └── Sequence length : 77

                                                    ALIGNED PU(S)
                                                    =============

PU 1      :GYFCESCRKIIQKLEDM
           ||||||||||||||||
TARGET    :DPMCSACEMAVVWMQNQ
ali. pos. 40               56
ori. pos. 1                17

PU 2      :VGPQPNEDTVTQAASQVCDKLKILRGLCK
             ||||::|||||||||||||:
TARGET    :LAQNKTQDLILDYVNQLCNRLP-------
ali. pos. 57                          85
ori. pos. 18                          46

PU 3      :KIMRSFLRRISWDILTGK
           ||||||||||||||||||
TARGET    :TIVSQYGQQILDLLLAET
ali. pos. 8                 25
ori. pos. 47                64

PU 4      :KPQAICVDIKICKE
           ||||||||||||
TARGET    :QPKKICSQVGLCA-
ali. pos. 26           39
ori. pos. 65           78

                                                    BEST ALIGNMENT
                                                    ==============

PUs       :             PU3              PU4              PU1                     PU2
ori. pos.:        ┌47              64┐┌65         78┐┌1             17┐┌18                         46┐
connect   :       +----------------++------------++---------------++----------------------------+
QUERY     :       KIMRSFLRRISWDILTGKKPQAICVDIKICKEGYFCESCRKIIQKLEDMVGPQPNEDTVTQAASQVCDKLKILRGLCK
match     :       ||||||||||||||||||||||||||||||  :||||||||||||||||  ||||::|||||||||||||:
TARGET    :VVSQECKTIVSQYGQQILDLLLAETQPKKICSQVGLCA-DPMCSACEMAVVWMQNQLAQNKTQDLILDYVNQLCNRLP-------
dist      :       1111011101100001101110000000117 2010000000000000007501112210111000110112
ali. pos.:          10        20        30        40        50        60        70        80


Aligned distance (match <=> dist): '|' <= 1 Å
                                   ':' <= 2 Å
                                   '.' <= 3 Å
```

Supplementary Fig. S6. Result of the alignment of d1nkl__ and d1qdma1 SCOP domains from the RIPC dataset by ICARUS. This output is an extract of the full terminal output given by ICARUS and represents the best alignment determined. ICARUS detects the circular permutation relating both structures as the third and fourth Protein Units (PUs) together are swapped

with PUs 1 and 2. The permutation occurs between two lysins at positions 46 and 47 of d1nkl__. This alignment also results in a high TM-score of 0.777. In bold and red are represented the residue pairs from the reference alignment that were correctly aligned, 67/72 in total, with a vast majority of matches < 1 Å.

```
 *  Score: 0.7
    Query: d1kiaa_
     ├── PU order:     PU1 |     PU2 |     PU3 |     PU4 |     PU5 |     PU6 |     PU7 |
     └── Regions :   18-35 |   36-57 |  58-115 | 116-175 | 176-244 | 245-260 | 261-292 |
    Target: d1nw5a_
     └── Sequence length : 270

                                                ALIGNED PU(S)
                                                =============


PU 1     :PDQYADGEAARVWQLYIG
           :... ..  .|||||||
TARGET   :DAVRE-PYDEETKAAYMK
ali. pos. 153              170
ori. pos. 18               35

PU 2     :D-TRSRTAEYKAWLLGLLRQHGC
            .:||||||:. :  :|  .
TARGET   :KRLNPESVEKGR-N--PTNVWR-
ali. pos. 172                   194
ori. pos. 36                    57


PU 3     :HRVLDVACGTGVDSIMLVEEGFSVTSVDASDKMLKYALKERWNRRKEPAFDKWVIEEA
          ||::||||||||::||||||||||:|||||||::||||:::. .  .::  |....
TARGET   :STVLDFFAGSGVTARVAIQEGRNSICTDAAPVFKEYYQKQLTFL--RSY--EIVEGAA
ali. pos. 245                                                      302
ori. pos. 58                                                       115

PU 4     :NWLTLDKDVPAGDGFDAVICLGNSFAHLP--DS--KGD-QSEHRLALKNIASMVRPGGLLVIDHR
          :::...:. |...|||||||.  :.|...   :  ||. .:|||||||||:|||||:|||||||.
TARGET   :DCLDTLAK-LPDDSVQLIICD--PPYNIMLADWDDHMDYIGWAKRWLAEAERVLSPTGSIAIFGG
ali. pos. 11                                                              75
ori. pos. 116                                                             175

PU 5     :NYDYILSTGCAPPGKNIYYK--S-DLTKDITTSVL-TVNNK----AHMVTLDYTVQVPGAGRDGAPGFSKFRLSYYP
          .:             .:::|  .  |||||||:.. .| ..    .  .:|:::::.: .     .:.
TARGET   :LQYQGEAGSGDLISIISHMRQNSKMLLANLIIWNYPNGMSAQRFFANRHEEIAWFAKTKK-----YFFDL-------
ali. pos. 76                                                                          152
ori. pos. 176                                                                         244

PU 6     :HCLASFTELVQEA-FGG
            ||||||||||| |:.
TARGET   :-KPAAVIERLVRALSHP
ali. pos. 227             243
ori. pos. 245             260

PU 7     :RCQHSVLGDFKPYRPGQAYVPCYFIHVLKKTG
                     :||:|||||:.||:
TARGET   :------M---SRLNGNSLERVGHPTQ------
ali. pos. 195                            226
ori. pos. 261                            292
```

```
                                    BEST ALIGNMENT
                                    ==============

PUs       :                                        PU4
ori. pos.:          ┌116                                                         175┐┌176
connect   :          +---------------------------------------------------------------++------------------------
QUERY     :          NWLTLDKDVPAGDGFDAVICLGNSFAHLP--DS--KGD-QSEHRLALKNIASMVRPGGLLVIDHRNYDYILSTGCAPPGKNIYYK--S-D
match     :          :::...:. |...|||||||.  :.|...   :  ||. .:||||||||:|||||:|||||||..:              .:::|  .
TARGET    :GTTRHVYDVCDCLDTLAK-LPDDSVQLIICD--PPYNIMLADWDDHMDYIGWAKRWLAEAERVLSPTGSIAIFGGLQYQGEAGSGDLISIISHMRQNSKM
dist      :          22233323 134311100013  231434  52  114 3211001111211100200001013428         98 942221  4 5
ali. pos.:         10        20        30        40        50        60        70        80        90

PUs       :           PU5                                            PU1                   PU2
ori. pos.:                                                244┐┌18            35┐ ┌36                 57┐┌261
connect   :---------------------------------------------------++----------------+ +---------------------++-----
QUERY     :LTKDITTSVL-TVNNK----AHMVTLDYTVQVPGAGRDGAPGFSKFRLSYYPPDQYADGEAARVWQLYIG-D-TRSRTAEYKAWLLGLLRQHGCRCQHSV
match     :|||||||:.. .| ..    .  .:|:::::.: .     .:.          :... ..  .|||||||   .:||||||:. :  :|  .
TARGET    :LLANLIIWNYPNGMSAQRFFANRHEEIAWFAKTKK-----YFFDL-------DAVRE-PYDEETKAAYMKDKRLNPESVEKGR-N--PTNVWR-------
dist      :0110011233 41534    355321222223254     4249        52333 336541000001 5 4211111123 2  215536
ali. pos.:         110       120       130       140       150       160       170       180       190

PUs       :       PU7                      PU6                                   PU3
ori. pos.:                      292┐┌245         260┐ ┌58                                                   11
connect   :-------------------------++---------------+ +-------------------------------------------------------
QUERY     :LGDFKPYRPGQAYVPCYFIHVLKKTGHCLASFTELVQEA-FGG-HRVLDVACGTGVDSIMLVEEGFSVTSVDASDKMLKYALKERWNRRKEPAFDKWVIE
match     :     :||:|||||:.||:         ||||||||||| |:. ||::||||||||::||||||||||:|||||||::||||:::. .  .::  |....
TARGET    :M---SRLNGNSLERVGHPTQ-------KPAAVIERLVRALSHPGSTVLDFFAGSGVTARVAIQEGRNSICTDAAPVFKEYYQKQLTFL--RSY--EIVEG
dist      :    8211211011231029       711011000000 123 01221111111122111100000021111110221111222453  422  13344
ali. pos.:         210       220       230       240       250       260       270       280       290

PUs       :
ori. pos.:5┐
connect   :-+
QUERY     :EA
match     :
TARGET    :AANFGAALQR
dist      :69
ali. pos.:         310

Aligned distance (match <=> dist): '|' <= 1 Å
                                   ':' <= 2 Å
                                   '.' <= 3 Å
```

Supplementary Fig. S7. Result of the alignment of d1kiaa_ and d1nw5a_ SCOP domains from the RIPC dataset by ICARUS. This output is an extract of the full terminal output given by ICARUS and represents the best alignment determined. Out of 12 residue pairs to correctly align, ICARUS successfully aligns 10, represented in bold and red font.

```
# ICARUS PSEUDOCODE

# Read input Protein1
# Read input Protein2
# Peel Protein1
# Peel Protein2

SET lvl = exploration level: {1, 2, 3, 4}
SET s = segmentation level (maximum number of PUs per exploration level):
    lvl 1 -> [2, 3]
    lvl 2 -> [4, 5]
    lvl 3 -> 6
    lvl 4 -> 7

PROCEDURE MAIN_PROCEDURE(ProteinA, ProteinB):
    IF no PUs available for ProteinA:
        Run TM-align(ProteinA, ProteinB)
    ELSE
        FOR each exploration level from 1 to lvl
            IF s level is available for ProteinA (enough number of PUs for asked lvl):
                Build tree alignment # exhaustive tree of all possible alignments
                    Nodes = alignments to do
                    Edges = actual alignment between connected nodes

                FOR each PU of segmentation level s to target # Run alignment tree
                    Align PU to ProteinA
                    Remove from ProteinA the regions where PUs are aligned for subsequent alignments

                    FOR each node at this lvl:
                        skip further exploration if successive alignment was already done once
            ELSE try with s - 1 while s > 2 (min s value)

        Merge all full branches (root to leaf) into pdb files.
        Every file represents a potential solution
        Calculate optimal TM-score of each solution

# A. Protein 1 vs. Protein 2
MAIN_PROCEDURE(Protein1, Protein2)

# B. Protein 2 vs. Protein 1
MAIN_PROCEDURE(Protein2, Protein1)

Gather all scores
Compute best global scores (multiple solutions possible)
Draw textual alignment
Delete intermediate files and clean tmp working directory
```

Supplementary Fig. S8. Pseudo code of ICARUS main algorithm.

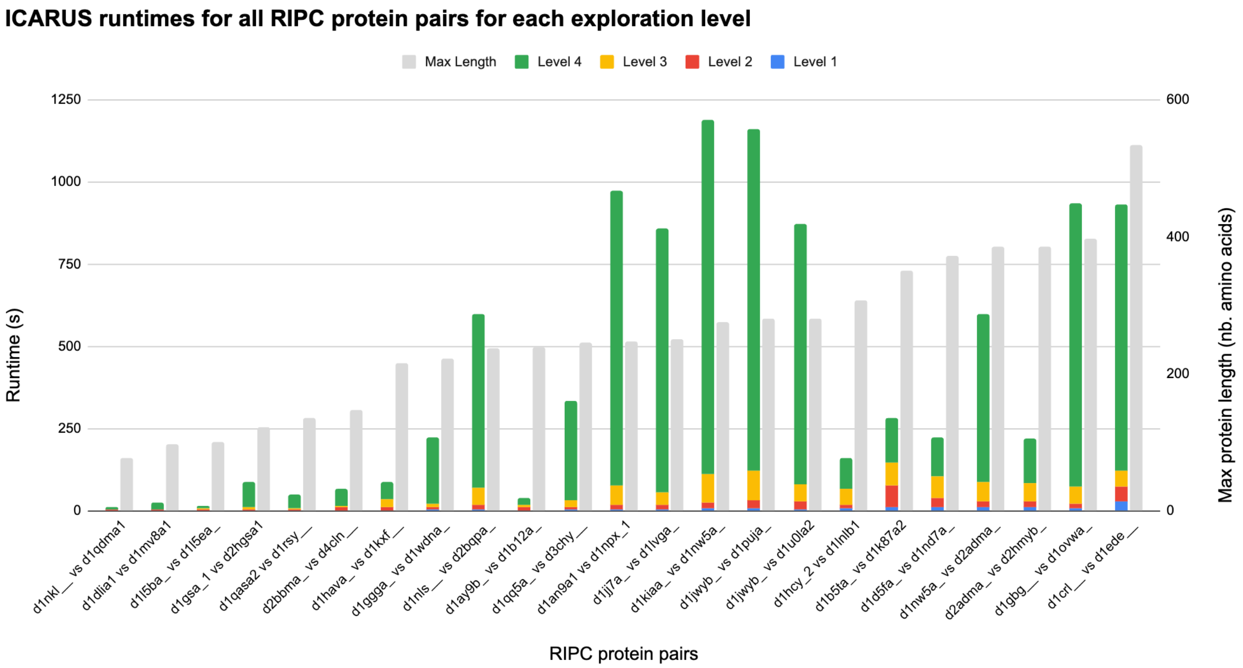

Supplementary Fig. S9. Runtimes (in seconds) of ICARUS exploration levels on the protein pairs of the RIPC dataset (only protein pairs which have a corresponding reference alignment available). Protein pairs are shown from left to right by growing maximum length (max length between each protein in pair). The longest runtime is 1078 seconds for d1kiaa_ vs d1nw5a_. The median runtime of level 1 is 5 s, of level 2 is 13 s, of level 3 is 46 s and level 4 is 136 seconds.

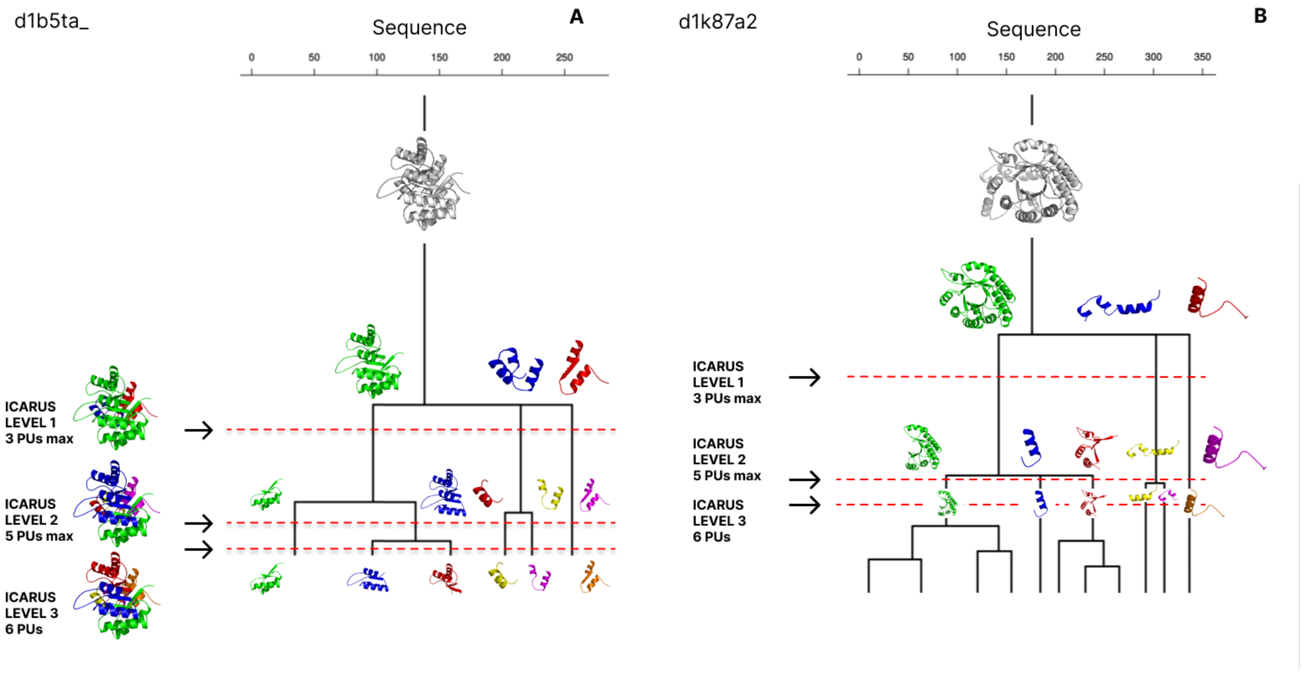

Supplementary Fig. S10. Illustration of Protein Peeling top-down clustering on the proteins d1b5ta_ (**A**) and d1k87a2 (**B**) from the RIPC dataset. ICARUS determines maximum exploration level 3 corresponding to a maximum of 6 PUs to align.

Supplementary Table S1. TM-scores of alignments for protein pairs of category C in the RIPC database (conformational variability) obtained by ICARUS, TM-align, FATCAT, DEDAL and KPAX. For ICARUS, results are given for each level of ICARUS exploration (from one to four). The best score is given in bold.

Protein 1	Protein 2	Length Protein 1	Length protein 2	ICARUS				TM-align	FATCAT	KPA X	DEDAL
				Level 1	Level 2	Level 3	Level 4				
d1dlia1	d1mv8a1	98	98	0.839	**0.872**	-	-	0.460	0.850	0.862	0.412
d1ggga_	d1wdna_	220	223	0.982	0.983	-	**0.984**	0.670	0.980	0.981	0.63
d1l5ba_	d1l5ea_	101	101	0.976	**0.978**	-	-	0.560	0.970	0.976	0.566
d2bbma_	d4cln__	148	148	0.851	0.882	**0.895**	-	0.430	0.820	0.892	0.441
			**MEAN**	0.912	0.929	0.895	**0.984**	0.530	0.905	0.928	0.512

Supplementary Table S2. TM-scores of alignments for protein pairs of category P in the RIPC database (circular permutations) obtained by ICARUS, TM-align, FATCAT, DEDAL and KPAX. For ICARUS, results are given for each level of exploration (from one to four). The best score is given in bold.

Protein 1	Protein 2	Length Protein 1	Length protein 2	ICARUS				TM-align	FATCA T	KPAX	DEDA L
				Level 1	Level 2	Level 3	Level 4				
d1nkl__	d1qdma1	78	77	0.638	**0.777**	-	-	0.520	0.520	0.73	0.502
d1nls__	d2bqpa_	237	228	0.912	0.918	**0.921**	**0.921**	0.490	0.490	0.492	0.488
d1qasa2	d1rsy__	126	135	0.756	0.761	0.762	-	0.720	0.730	**0.79**	0.718
d1d5ra1	d1rsy__	133	135	0.654	**0.691**	0.682	-	0.560	0.630	0.635	0.502
			**MEAN**	0.740	0.787	0.788	**0.921**	0.573	0.593	0.662	0.504

Supplementary Table S3. TM-scores of alignments for protein pairs of PI category in the RIPC database (circular permutations associated with insertions) obtained by ICARUS, TM-align, FATCAT, DEDAL and KPAX. For ICARUS, results are given for each level of exploration (from one to four). The best score is given in bold.

Protein 1	Protein 2	Length Protein 1	Length protein 2	ICARUS				TM-align	FATCA T	KPAX	DEDA L
				Level 1	Level 2	Level 3	Level 4				
d1jwyb_	d1puja_	281	261	0.562	0.628	0.669	**0.695**	0.310	0.570	0.355	0.334
d1jwyb_	d1u0la2	281	212	0.575	0.655	0.702	**0.729**	0.340	0.580	0.436	0.332
d1kiaa_	d1nw5a_	275	270	0.614	0.629	0.671	**0.7**	0.360	0.440	0.386	0.355
d1nw5a_	d2adma_	270	385	0.591	0.635	0.683	**0.71**	0.370	0.640	0.688	0.359
d1qq5a_	d3chy__	245	128	0.645	0.785	0.768	**0.797**	0.490	0.540	0.756	0.558
			**MEAN**	0.597	0.666	0.699	**0.726**	0.374	0.554	0.524	0.468

Supplementary Table S4. TM-scores of alignments for protein pairs of category I of the RIPC database (insertions/deletions) obtained by ICARUS, TM-align, FATCAT, DEDAL and KPAX. For ICARUS, results are given for each level of exploration (from one to four). The best score is given in bold.

Protein 1	Protein 2	Length Protein 1	Length protein 2	ICARUS				TM-align	FATCAT	KPAX	DEDA L
				Level 1	Level 2	Level 3	Level 4				
d1an9a1	d1npx_1	247	198	0.602	0.644	0.677	**0.709**	0.570	0.600	0.592	0.571
d1ay9b_	d1b12a_	108	239	0.69	0.768	0.776	**0.782**	0.680	0.680	0.702	0.662
d1crl__	d1ede__	534	310	0.614	0.669	0.708	0.73	0.590	**0.750**	0.727	0.595
d1gbg__	d1ovwa_	214	397	0.76	0.77	0.706	**0.833**	0.750	0.750	0.808	0.747
d1hcy_2	d1lnlb1	263	307	0.579	0.593	0.606	0.648	0.510	0.590	**0.659**	0.506
d2adma_	d2hmyb_	385	327	0.552	0.613	0.643	0.662	0.400	0.630	**0.677**	0.405
d1aqza_	d1a2pa_	142	108	0.559	0.599	-	-	0.480	0.470	**0.665**	0.436
d1b09a_	d1dy4a_	206	433	0.607	0.681	0.635	**0.724**	0.590	0.400	0.62	0.587
d1dy4a_	d2sak__	88	121	0.514	0.671	-	**0.694**	0.530	0.520	0.55	0.493
d1ed9a_	d1p49a_	449	548	0.531	0.569	0.57	**0.573**	0.490	0.540	0.53	0.499
d1hx6a1	d1p2za2	230	312	0.66	0.654	0.721	**0.745**	0.460	0.480	0.524	0.461
d1ng4a2	d3cox_2	88	130	0.742	0.736	**0.829**	-	0.570	0.570	0.633	0.513
			**MEAN**	0.618	0.664	0.687	**0.710**	0.552	0.582	0.641	0.508

Supplementary Table S5. TM-scores of alignments obtained by ICARUS, TM-align, FATCAT, DEDAL and KPAX and for each level of ICARUS exploration (from one to four) for IC protein pairs (insertions and associated conformational variability). The best score is presented in bold.

Protein 1	Protein 2	Length Protein 1	Length protein 2	ICARUS				TM-align	FATCAT	KPAX	DEDAL
				Level 1	Level 2	Level 3	Level 4				
d1d5fa_	d1nd7a_	350	373	0.851	0.915	0.92	0.926	0.590	0.860	**0.944**	0.6
d1hava_	d1kxf__	216	159	0.662	**0.698**	0.681	-	0.640	0.620	0.691	0.639
d1jj7a_	d1lvga_	251	190	0.463	0.589	0.617	**0.664**	0.420	0.490	0.6	0.374
d1adl__	d1mup__	131	157	0.659	**0.713**	**0.713**	-	0.530	0.530	0.604	0.532
d1dmaa_	d1lt3a_	204	226	0.477	0.513	0.594	**0.62**	0.390	0.420	0.45	0.395
d1lzy__	d148le_	129	162	0.562	**0.799**	-	-	0.440	0.510	0.591	0.426
			**MEAN**	0.612	0.705	0.705	**0.737**	0.502	0.572	0.647	0.497

Supplementary Table S6. TM-scores of alignments for protein pairs of category IR in the RIPC database (insertions and associated repeats) obtained by ICARUS, TM-align, FATCAT and KPAX, DEDAL. For ICARUS, results are given for each level of exploration (from one to four). The best score is given in bold.

Protein 1	Protein 2	Length Protein 1	Length protein 2	ICARUS				TM-align	FATCAT	KPAX	DEDAL
				Level 1	Level 2	Level 3	Level 4				
d1afra_	d1jkua_	345	266	0.649	0.721	0.708	**0.73**	0.530	0.650	0.530	0.54
d1cpo_1	d1cpo_2	119	179	0.554	0.647	0.67	**0.704**	0.450	0.440	0.450	0.434
d1he9a_	d1nfn__	134	132	0.604	0.67	0.778	**0.811**	0.500	0.640	0.500	0.481
d1olza2	d2trcb_	474	340	0.714	0.778	0.803	**0.816**	0.710	0.730	0.710	0.708
d1xyza_	d2hvm__	320	273	0.697	0.707	0.723	**0.731**	0.640	0.640	0.640	0.632
			**MEAN**	0.644	0.705	0.736	**0.758**	0.566	0.620	0.566	0.511

Supplementary Table S7. TM-scores of alignments for protein pairs of CP category in the RIPC database (circular permutations associated with conformational variability) obtained by ICARUS, TM-align, FATCAT, DEDAL and KPAX. For ICARUS, results are given for each level of exploration (from one to four). The best score is given in bold.

Protein 1	Protein 2	Length Protein 1	Length protein 2	ICARUS				TM-align	FATCAT	KPAX	DEDAL
				Level 1	Level 2	Level 3	Level 4				
d1gsa_1	d2hgsa1	122	102	0.652	0.732	**0.738**	-	0.660	0.650	0.703	0.589
d1b6a_1	d1bia_1	74	63	0.642	0.675	-	-	0.510	0.580	**0.683**	0.51
			**MEAN**	0.647	0.704	**0.738**	-	0.585	0.615	0.693	0.489

Supplementary Table S8. TM-scores of alignments for protein pairs in the PR category of the RIPC database (circular permutations and associated repeats) obtained by ICARUS, TM-align, FATCAT, DEDAL and KPAX. For ICARUS, results are given for each level of exploration (from one to four). The best score is given in bold.

Protein 1	Protein 2	Length Protein 1	Length protein 2	ICARUS				TM-align	FATCAT	KPAX	DEDAL
				Level 1	Level 2	Level 3	Level 4				
d1b5ta_	d1k87a2	275	351	0.6525	0.707	0.746	NA	0.560	**0.750**	0.687	0.542

Supplementary Table S9. TM-scores of alignments for protein pairs in the CR category of the RIPC database (conformational variability and associated repeats) obtained by ICARUS, TM-align, FATCAT, DEDAL and KPAX. For ICARUS, results are given for each level of exploration (from one to four). The best score is given in bold.

Protein 1	Protein 2	Length Protein 1	Length protein 2	ICARUS				TM-align	FATCAT	KPAX	DEDAL
				Level 1	Level 2	Level 3	Level 4				
d1aj3__	d2spca_	98	107	0.824	0.887	**0.897**	NA	0.530	0.780	0.780	NA

Supplementary Table S10. Global mean TM-scores of alignments for protein pairs obtained by ICARUS, TM-align, FATCAT, DEDAL and KPAX on the full RIPC dataset. For ICARUS, results are given for each level of exploration (from one to four). The best global mean score is given in bold.

ICARUS				TM-align	FATCAT	KPAX	DEDAL
**Level 1**	**Level 2**	**Level 3**	**Level 4**				
0.675	0.728	0.747	**0.759**	0.626	0.516	0.676	0.525

Supplementary Table S11. Detailed performances of each method on the 23 reference alignments provided by the RIPC dataset in terms of percentage of accurately aligned positions according to the reference alignment positions. **Struct. Rel.** represent the difficulty class, combination of R (Repetition), I (InDels), P (Permutation) and C (Conformational variability).

				ICARUS								
**Protein 1**	**Protein 2**	**Struct. Rel.**	**Nb. ref. residues**	**Level 1**	**Level 2**	**Level 3**	**Level 4**	**TM-align**	**FATCAT**	**DEDAL**	**KPAX**	**PAUL**
d1an9a1	d1npx_1	I	11	54.5	63.6	63.6	63.6	**100**	63.6	81.8	72.7	63.6
d1ay9b_	d1b12a_	I	10	**100**	90	90	90	**100**	90	**100**	**100**	90
d1b5ta_	d1k87a2	R P	8	**50**	**50**	**50**	**50**	**50**	0	0	**50**	50
d1crl__	d1ede__	I	3	66.7	**100**	33.3	33.3	**100**	66.7	**100**	**100**	66.7
d1d5fa_	d1nd7a_	I C	6	**100**	**100**	**100**	83.3	66.7	**100**	66.7	**100**	**100**
d1dlia1	d1mv8a1	C	4	**100**	**100**	**100**	**100**	50	**100**	50	**100**	**100**
d1gbg__	d1ovwa_	I	3	**100**	66.7	66.7	66.7	0	33.3	33.3	**100**	**100**
d1ggga_	d1wdna_	C	220	**96.8**	**96.8**	**96.8**	**96.8**	73.6	**96.8**	73.6	**96.8**	**97.3**
d1gsa_1	d2hgsa1	P C	5	40	40	**100**	**100**	0	40	80	40	**80**
d1hava_	d1kxf__	I C	4	**100**	**100**	**100**	**100**	**100**	25	**100**	**100**	**100**
d1hcy_2	d1lnlb1	I	4	50	50	25	25	**100**	50	50	50	50
d1jj7a_	d1lvga_	I C	8	**100**	**100**	**100**	0	50	**100**	**100**	**100**	**100**
d1jwyb_	d1puja_	I P	12	75	75	**66.7**	25	33.3	0	33.3	33.3	0
d1jwyb_	d1u0la2	I P	11	**100**	**100**	90.9	27.3	36.4	0	36.4	36.4	0
d1kiaa_	d1nw5a_	I P	12	83.3	83.3	83.3	83.3	25	25	58.3	25	0
d1l5ba_	d1l5ea_	C	101	**100**	**100**	**100**	**100**	72.3	**100**	72.3	**100**	**100**
d1nkl__	d1qdma1	P	72	0	**93.1**	**93.1**	**93.1**	0	0	48.6	0	83.3
d1nls__	d2bqpa_	P	6	83.3	83.3	83.3	83.3	**100**	83.3	83.3	83.3	30.8
d1nw5a_	d2adma_	I P	13	**100**	84.6	69.2	69.2	0	46.2	38.5	46.2	84
d1qasa2	d1rsy__	P	75	90.7	90.7	90.7	90.7	**100**	84	84	82.7	0
d1qq5a_	d3chy__	I P	3	0	33.3	33.3	33.3	0	0	**66.7**	0	**100**
d2adma_	d2hmyb_	I	12	**100**	66.7	**100**	66.7	**100**	**100**	**100**	83.3	**100**
d2bbma_	d4cln__	C	148	95.9	**100**	**100**	**100**	**100**	97.3	89.2	96.6	63.6
			**MEAN**	77.7	**81.2**	79.8	68.7	57.7	56.6	67.2	69.4	66.7

Supplementary Table. S12. P-values of Wilcoxon paired signed-rank tests executed on performances of each ICARUS exploration level against all concurrent methods on the reference alignments of the RIPC dataset. Null hypothesis is that the average agreements of ICARUS exploration levels with the reference alignment are equal to other methods. Alternative hypothesis is that ICARUS average agreement performances are significantly higher than other methods. In bold, P-values lower than the significant level of 0.1.

	KPAX	FATCAT	DEDAL	TMalign
**ICARUS L1**	**7.76E-2**	**5.62E-3**	1.01E-1	**2.46E-2**
**ICARUS L2**	**5.39E-2**	**1.85E-3**	**1.83E-2**	**7.85E-3**
**ICARUS L3**	**9.10E-2**	**4.58E-3**	**2.42E-2**	**2.67E-2**
**ICARUS L4**	6.82E-1	**6.49E-2**	2.66E-1	2.00E-1

Supplementary Table. S13. P-values of paired t-tests executed on performances of concurrent methods against ICARUS exploration level 1 (giving best performances compared to other levels) on the reference alignments of the SISY dataset. Null hypothesis is that the average agreements of concurrent methods with the reference alignment are equal to ICARUS exploration level 1 (best performing level on SYSI). Alternative hypothesis is that concurrent methods average agreement performances are significantly greater than ICARUS Level 1. Significant p-values are in bold. Apart from DEDAL, the p-values > α (α = 0.05 and even 0.1), for those methods H0 can not be rejected. Hence, apart from DEDAL, other concurrent methods do not perform significantly better than ICARUS Level 1 on the reference alignments of the SISY dataset.

	ICARUS L1
**KPAX**	2.02E-1
**FATCAT**	6.30E-1
**DEDAL**	**7.14E-3**
**TMalign**	8.71E-1

Supplementary Table. S14. P-values of Wilcoxon paired signed-rank tests executed on performances of ICARUS exploration level 4 (best performing on SYSI dataset) against all concurrent methods in terms of TM-score. Null hypothesis is that the average TM-scores of ICARUS L4 are equal to other methods. Alternative hypothesis is that ICARUS average average TM-scores are significantly higher than other methods. In bold, P-values lower than the significant level of 0.0.5. ICARUS generates significantly higher TM-scores on average than concurrent methods on the SYSI dataset.

	KPAX	FATCAT	DEDAL	TMalign
**ICARUS L4**	**4.81E-4**	**< 2.2e-16**	**< 2.2e-16**	**< 2.2e-16**

Supplementary Table S15. Detailed performances of each method on the 111 reference alignments provided by the SISY dataset. **TM** corresponds to the best TM-scores obtained after aligning both proteins (1 vs. 2 or 2 vs. 1), **res. pairs** correspond to the percentage of residues correctly aligned according to reference alignments, and **Alt.** refers to the index of the alternative alignment, if available, that gave the best result, else ”NA”. In bold the highest mean percentage of residues correctly aligned according to reference alignments, and the highest mean TM-scores obtained. TM-align does not support alignment of proteins composed of multiple chains.

		**ICARUS**																							
**PROTEINS**		**ICARUS L1**			**ICARUS L2**			**ICARUS L3**			**ICARUS L4**			**FATCAT**			**DEDAL**			**KPAX**			**TMalign**		
		**TM**	**res. pairs**	**Alt.**	**TM**	**res. pairs**	**Alt.**	**TM**	**res. pairs**	**Alt.**	**TM**	**res. pairs**	**Alt.**	**TM**	**res. pairs**	**Alt.**	**TM**	**res. pairs**	**Alt.**	**TM**	**res. pairs**	**Alt.**	**TM**	**res. pairs**	**Alt.**
**1s0uA**	**1vloA**	0.457	0	NA	0.54	0.188	NA	0.54	0.188	NA	0.592	0.188	NA	0.528	0	NA	0.245	0	NA	0.504	0.146	NA	0.271	0	NA
**1r5bA**	**1s0uA**	0.727	1	NA	0.731	1	NA	0.74	1	NA	0.768	1	NA	0.719	0.902	NA	0.462	0.902	NA	0.754	0.967	NA	0.473	0.59	NA
**1g60A**	**1pjzA**	0.659	1	NA	0.679	1	NA	0.698	1	NA	0.723	1	NA	0.544	0	NA	0.386	0	NA	0.568	0	NA	0.416	0	NA
**2f6uA**	**2tpsA**	0.679	0	NA	0.707	0	NA	0.718	0	NA	0.741	0	NA	0.654	0	NA	0.643	0	NA	0.739	0	NA	0.653	0	NA
**1iapA**	**1zv4X**	0.735	0.83	NA	0.839	0.58	NA	0.863	0.727	NA	0.885	0.352	NA	0.69	0.955	NA	0.697	0.977	NA	0.792	0.909	NA	0.704	0.966	NA
**1hx1B**	**1ugoA**	0.742	0.69	NA	0.766	0.338	NA	0.766	0.338	NA	0.766	0.338	NA	0.668	1	NA	0.7	1	NA	0.787	0.958	NA	0.707	1	NA
**1yadA**	**2tpsA**	0.898	1	NA	0.907	1	NA	0.927	1	NA	0.931	0.94	NA	0.881	1	NA	0.881	1	NA	0.938	0.976	NA	0.882	1	NA
**1ej0A**	**1qamA**	0.682	0.817	NA	0.744	0.859	NA	0.789	0.465	NA	0.801	0.563	NA	0.638	0.732	NA	0.638	0.831	NA	0.752	0.873	NA	0.651	0.746	NA
**1tv5A**	**1vizA**	0.726	0.788	NA	0.761	0.534	NA	0.799	0.373	NA	0.802	0.669	NA	0.671	0.754	NA	0.661	0.686	NA	0.821	0.771	NA	0.667	0.695	NA
**1fohA**	**2gb0B**	0.656	0.94	NA	0.678	0.94	NA	0.721	0.93	NA	0.737	0.93	NA	0.651	0.7	NA	0.627	0.93	NA	0.692	0.76	NA	0.636	0.94	NA
**1ezvA**	**1ie0A**	0.598	0.203	2	0.698	0.22	2	0.713	0.22	2	0.768	0.22	2	0.596	0.153	2	0.591	0.407	2	0.754	0.237	2	0.61	0.39	2
**1ezvA**	**1ezvB**	0.875	0.986	4	0.885	0.986	4	0.89	1	4	0.89	1	4	0.869	0.986	4	0.866	0.986	4	0.909	0.917	1	0.869	0.986	4
**1gl0I**	**1pmcA**	0.479	1	NA	0.479	1	NA	0.479	1	NA	0.479	1	NA	0.407	1	NA	0.417	1	NA	0.639	1	NA	0.458	1	NA
**1egaB**	**1j5eC**	0.529	0.898	NA	0.686	0.898	NA	0.727	0.898	NA	0.778	0.763	NA	0.396	0.932	NA	0.379	0.695	NA	0.48	0.932	NA	0.353	0	NA
**1dnlA**	**1rfeA**	0.706	0.868	NA	0.774	0.855	NA	0.778	0.855	NA	0.789	0.855	NA	0.711	0.868	NA	0.66	0.934	NA	0.83	0.855	NA	0.657	0.868	NA
**1bx4A**	**1lioA**	0.907	1	NA	0.913	1	NA	0.913	1	NA	0.935	1	NA	0.843	0.909	NA	0.838	0.951	NA	0.957	0.994	NA	0.856	0.883	NA
**1ec6A**	**2fmrA**	0.686	0.667	NA	0.752	0.632	NA	0.752	0.632	NA	0.752	0.632	NA	0.587	0.86	NA	0.621	0.86	NA	0.755	0.895	NA	0.627	0.86	NA
**1l0bA**	**1l7bA**	0.655	0	1	0.687	0	1	0.796	0	1	0.796	0	1	0.606	0	1	0.52	0	1	0.663	0	1	0.525	0	1
**2nacA**	**3gpdG**	0.491	0.628	NA	0.531	0.628	NA	0.567	0.628	NA	0.567	0.628	NA	0.347	0.526	NA	0.324	0.628	NA	0.351	0.718	NA	0.341	0	NA

**1jclA**	**1n7kA**	0.877	0.959	NA	0.89	0.959	NA	0.905	0.832	NA	0.92	0.832	NA	0.861	0.995	NA	0.863	1	NA	0.93	0.995	NA	0.863	1	NA
**1r5pA**	**1t4yA**	0.655	1	NA	0.699	0.786	NA	0.699	0.786	NA	0.699	0.786	NA	0.587	1	NA	0.436	1	NA	0.682	0.929	NA	0.475	1	NA
**1ie0A**	**1j6wA**	0.88	0.934	NA	0.894	0.934	NA	0.897	0.912	NA	0.898	0.912	NA	0.843	0.942	NA	0.889	1	NA	0.895	0.927	NA	0.854	0.942	NA
**1jhdA**	**1sgvA**	0.39	0.725	NA	0.572	0.65	NA	0.658	0.725	NA	0.685	0.725	NA	0.525	0	NA	0.236	0.725	NA	0.24	0.65	NA	0.296	0	NA
**1b10A**	**1i17A**	0.662	0.912	NA	0.69	0.647	NA	0.711	0.456	NA	0.711	0.456	NA	0.619	0.956	NA	0.623	0.956	NA	0.815	0.912	NA	0.635	0.956	NA
**1wv3A**	**2g1lA**	0.705	0.745	2	0.754	0.809	2	0.785	0.809	2	0.785	0.809	2	0.638	0.83	2	0.655	0.872	2	0.738	0.787	2	0.647	0.851	2
**1i6vE**	**1qklA**	0.482	0.818	NA	0.555	0.364	NA	0.615	0.545	NA	0.615	0.545	NA	0.368	1	NA	0.367	0.909	NA	0.34	0.455	NA	0.371	0.909	NA
**1auaA**	**1vc1A**	0.655	0.06	NA	0.741	0.024	NA	0.766	0.024	NA	0.766	0.024	NA	0.708	0.301	NA	0.561	0.072	NA	0.806	0.349	NA	0.581	0.072	NA
**1cr5A**	**3engA**	0.459	0.974	NA	0.539	0	NA	0.599	0.692	NA	0.599	0.692	NA	0.464	0.821	NA	0.383	0.974	NA	0.479	0.897	NA	0.386	1	NA
**1n10A**	**3engA**	0.493	0	NA	0.569	0	NA	0.611	0	NA	0.611	0	NA	0.492	0	NA	0.428	0	NA	0.551	0	NA	0.452	0	NA
**1fc9A**	**1ky9B**	0.465	0.855	NA	0.479	0.87	NA	0.544	0.783	NA	0.544	0.783	NA	0.406	0.87	NA	0.271	0.942	NA	0.447	0.928	NA	0.289	0.101	NA
**1r9cA**	**1xy7B**	0.729	1	1	0.787	1	1	0.788	1	1	0.788	1	1	0.683	0.833	4	0.706	1	1	0.77	1	1	0.719	1	1
**1orbA**	**1qxnA**	0.738	0.909	2	0.79	0.894	2	0.79	0.894	2	0.832	0.258	2	0.61	0.5	1	0.685	0.53	1	0.83	0.833	2	0.696	0.818	2
**1d4dA**	**1jnrA**	0.664	0.941	NA	0.687	0.941	NA	0.693	0.941	NA	0.695	0.893	NA	0.629	0.946	NA	0.608	0.941	NA	0.683	0.951	NA	0.608	0.941	NA
**1ukvG**	**1vg9A**	0.896	0.979	NA	0.898	0.979	NA	0.904	0.975	NA	0.905	0.975	NA	0.858	0.982	NA	0.862	0.986	NA	0.929	0.954	NA	0.862	0.986	NA
**1j5pA**	**1ps9A**	0.605	1	1	0.694	1	2	0.778	1	2	0.796	1	2	0.476	0	1	0.341	1	1	0.389	1	1	0.424	0	1
**1j5xA**	**1nriA**	0.609	0.891	1	0.741	0.719	1	0.753	0.719	1	0.78	0.719	1	0.637	0.891	1	0.532	0.984	2	0.651	0.781	1	0.541	0.891	1
**1r18A**	**1vbfA**	0.8	0.917	NA	0.802	0.94	NA	0.806	0.917	NA	0.82	0.94	NA	0.764	0.976	NA	0.769	1	NA	0.79	0.964	NA	0.764	0.976	NA
**1olzA**	**1shyB**	0.768	0.947	NA	0.781	0.947	NA	0.781	0.947	NA	0.795	0.947	NA	0.759	0.789	NA	0.745	0.579	NA	0.784	1	NA	0.747	0.579	NA
**1rieA**	**1zinA**	0.462	0	NA	0.562	0	NA	0.61	0	NA	0.654	0	NA	0.358	0	NA	0.17	0.545	NA	0.56	0	NA	0.308	0	NA
**1pohA**	**1sphA**	0.909	1	NA	0.944	1	NA	0.944	1	NA	0.944	1	NA	0.889	1	NA	0.889	1	NA	0.923	1	NA	0.89	1	NA
**1y8qA**	**1zfnA**	0.87	1	NA	0.88	0.989	NA	0.882	0.989	NA	0.918	0.989	NA	0.854	1	NA	0.853	1	NA	0.907	1	NA	0.854	1	NA
**1j5yA**	**1pchA**	0.615	0.682	NA	0.752	0.318	NA	0.752	0.318	NA	0.752	0.318	NA	0.601	0.682	NA	0.603	0.879	NA	0.758	0.591	NA	0.609	0.652	NA
**1f33A**	**1j30B**	0.828	0.981	NA	0.828	0.981	NA	0.828	0.981	NA	0.828	0.981	NA	0.768	0.491	NA	0.472	0.491	NA	0.906	0.991	NA	0.473	0.491	NA

**1d00A**	**1k2fA**	0.634	0.988	NA	0.64	0.942	NA	0.64	0.942	NA	0.683	0.733	NA	0.577	0.93	NA	0.579	0.988	NA	0.628	0.988	NA	0.586	0.988	NA
**1eu1A**	**1qcsA**	0.57	0.941	NA	0.702	0.941	NA	0.737	0.941	NA	0.737	0.941	NA	0.382	0.941	NA	0.379	1	NA	0.409	0.98	NA	0.378	0.549	NA
**1iw4A**	**1lr7A**	0.57	0	NA	0.57	0	NA	0.57	0	NA	0.57	0	NA	0.326	0.472	NA	0.426	0.556	NA	0.579	0.694	NA	0.451	0.306	NA
**1it8A**	**2ashA**	0.863	0.983	NA	0.91	0.977	NA	0.911	0.973	NA	0.912	0.973	NA	0.841	0.983	NA	0.84	0.983	NA	0.877	0.97	NA	0.841	0.983	NA
**1jr1A**	**2bleA**	0.899	0.942	NA	0.899	0.942	NA	0.899	0.942	NA	0.899	0.942	NA	0.877	0.979	NA	0.884	0.979	NA	0.924	0.921	NA	0.885	0.979	NA
**1h0zA**	**1hdlA**	0.59	0.385	NA	0.59	0.385	NA	0.59	0.385	NA	0.59	0.385	NA	0.467	0.974	NA	0.456	0.974	NA	0.617	0.667	NA	0.49	0.923	NA
**1awwA**	**1gxiE**	0.491	0.724	NA	0.564	0.69	NA	0.564	0.69	NA	0.564	0.69	NA	0.515	0.966	NA	0.531	1	NA	0.64	1	NA	0.535	0.966	NA
**1qf6A**	**1v7oA**	0.858	0.976	NA	0.872	0.8	NA	0.873	0.8	NA	0.873	0.8	NA	0.836	0.984	NA	0.828	0.984	NA	0.878	0.984	NA	0.838	0.984	NA
**1omhA**	**1r9wA**	0.597	0	NA	0.705	0	NA	0.754	0	NA	0.763	0	NA	0.603	0.392	NA	0.379	0.608	NA	0.697	0.647	NA	0.394	0.608	NA
**1h9mA**	**1q12A**	0.758	1	NA	0.809	0.79	NA	0.815	0.704	NA	0.832	0.617	NA	0.717	0.889	NA	0.72	0	NA	0.782	0.877	NA	0.727	0.877	NA
**1qftA**	**1u0xA**	0.675	0.671	NA	0.697	0.38	NA	0.698	0.696	NA	0.729	0.696	NA	0.663	0.608	NA	0.654	0.608	NA	0.772	0.57	NA	0.662	0.544	NA
**1szwA**	**2apoA**	0.559	0.913	NA	0.578	0.913	NA	0.598	0.884	NA	0.618	0.913	NA	0.49	0.536	NA	0.39	0.913	NA	0.443	0.913	NA	0.391	0.899	NA
**1lw3A**	**1r6hA**	0.672	0.731	NA	0.715	0.164	NA	0.787	0.582	NA	0.809	0.582	NA	0.676	0.791	NA	0.652	0.716	NA	0.703	0.537	NA	0.663	0.731	NA
**1oa8A**	**1v06A**	0.747	0.986	NA	0.765	1	NA	0.765	1	NA	0.765	1	NA	0.761	1	NA	0.556	1	NA	0.819	0.986	NA	0.557	1	NA
**1nn4A**	**1uslA**	0.895	0.874	NA	0.898	0.874	NA	0.898	0.874	NA	0.898	0.874	NA	0.886	1	NA	0.886	1	NA	0.898	0.972	NA	0.882	1	NA
**1nw6A**	**1xvaA**	0.622	0.891	NA	0.643	0.845	NA	0.659	0.783	NA	0.681	0.783	NA	0.258	0.318	NA	0.357	0.527	NA	0.389	0.566	NA	0.358	0	NA
**1jvnA**	**1uwcA**	0.475	0	NA	0.604	0	NA	0.624	0	NA	0.655	0	NA	0.554	0	NA	0.387	0	NA	0.489	0	NA	0.401	0	NA
**1l3aA**	**1pcfA**	0.729	0	1	0.781	0	1	0.781	0	1	0.781	0	1	0.523	1	2	0.438	0.7	1	0.781	0.525	2	0.548	0.85	2
**1v7oA**	**1vjeA**	0.587	0.195	NA	0.624	0.416	NA	0.669	0.221	NA	0.669	0.221	NA	0.496	0.532	NA	0.424	0.532	NA	0.564	0.558	NA	0.467	0.195	NA
**1c7qA**	**1gzdA**	0.872	0.914	NA	0.876	0.86	NA	0.879	0.86	NA	0.886	0.847	NA	0.867	0.949	NA	0.867	0.952	NA	0.899	0.919	NA	0.867	0.949	NA
**1mk5A**	**1wbiA**	0.854	0.971	NA	0.863	0.99	NA	0.863	0.99	NA	0.863	0.99	NA	0.832	0.924	NA	0.846	0.981	NA	0.877	0.99	NA	0.847	0.971	NA
**1kncA**	**2gmyA**	0.755	0.868	1	0.846	0.868	2	0.846	0.868	2	0.875	0.868	2	0.731	0.981	1	0.741	0.981	1	0.85	0.925	1	0.742	0.981	1
**1fybA**	**1pjuA**	0.749	1	1	0.78	1	1	0.781	1	1	0.781	1	1	0.631	1	1	0.363	1	3	0.645	1	1	0.402	1	1
**1hkyA**	**1i8nA**	0.611	1	NA	0.636	1	NA	0.665	1	NA	0.665	1	NA	0.554	0.882	NA	0.535	0.941	NA	0.707	1	NA	0.554	1	NA

**1ecmA**	**2csmA**	0.867	0.548	2	0.9	0.435	2	0.913	0	1	0.924	0	1	0.828	0.968	2	0.83	0.935	2	0.921	0.403	2	0.66	0.548	2
**1i8nA**	**2hgfA**	0.662	0.984	NA	0.705	0.623	NA	0.705	0.623	NA	0.705	0.623	NA	0.618	0.918	NA	0.629	1	NA	0.701	0.984	NA	0.633	1	NA
**1fyeA**	**1q7rA**	0.601	0.667	NA	0.629	0.667	NA	0.741	0.587	NA	0.78	0.762	NA	0.562	0.667	NA	0.539	0.698	NA	0.618	0.667	NA	0.538	0.667	NA
**1dqxA**	**1v5xA**	0.737	0.513	NA	0.806	0.204	NA	0.806	0.204	NA	0.816	0.062	NA	0.7	0.593	NA	0.693	0.593	NA	0.808	0.584	NA	0.702	0.619	NA
**1s3sE**	**1wlfA**	0.842	1	1	0.85	1	1	0.855	1	1	0.857	1	4	0.782	1	1	0.765	0.969	1	0.914	1	1	0.783	1	1
**1booA**	**1nw6A**	0.808	0.984	NA	0.809	0.989	NA	0.809	0.989	NA	0.825	0.989	NA	0.749	1	NA	0.755	1	NA	0.809	0.995	NA	0.756	0.995	NA
**1pe31**	**2pspA**	0.665	0.762	2	0.721	0.881	2	0.734	0.738	1	0.739	0.5	2	0.58	1	1	0.575	1	2	0.67	1	1	0.582	1	1
**1efcA**	**1vloA**	0.436	0.565	2	0.538	0.565	2	0.563	0.609	2	0.61	0.565	2	0.434	0.783	1	0.223	0.87	1	0.492	0.609	2	0.306	0	1
**1b12A**	**2mysA**	0.432	0	NA	0.486	0	NA	0.54	0	NA	0.591	0	NA	0.366	0	NA	0.264	0.972	NA	0.421	0	NA	0.363	0	NA
**1b56A**	**2ft9A**	0.901	1	NA	0.915	1	NA	0.915	1	NA	0.915	1	NA	0.871	0.991	NA	0.871	0.991	NA	0.94	1	NA	0.871	0.991	NA
**1h9cA**	**1phrA**	0.802	0.639	NA	0.813	0.554	NA	0.813	0.554	NA	0.813	0.554	NA	0.768	0.747	NA	0.684	0.783	NA	0.874	0.639	NA	0.689	0.783	NA
**1hmsA**	**1smpI**	0.656	0.476	NA	0.681	0	NA	0.681	0	NA	0.681	0	NA	0.493	0.024	NA	0.485	0.071	NA	0.735	0.357	NA	0.463	0.024	NA
**1omhA**	**1p4dA**	0.842	0.925	NA	0.842	0.925	NA	0.848	0.953	NA	0.856	0.925	NA	0.775	0.93	NA	0.793	0.972	NA	0.809	0.916	NA	0.783	0.953	NA
**1l9lA**	**1qdmA**	0.773	0.554	NA	0.852	0.536	NA	0.852	0.536	NA	0.852	0.536	NA	0.469	0	NA	0.535	0.464	NA	0.731	0	NA	0.543	0	NA
**1otrA**	**2di0A**	0.751	1	NA	0.818	1	NA	0.818	1	NA	0.818	1	NA	0.66	1	NA	0.66	1	NA	0.773	1	NA	0.688	1	NA
**1qftA**	**1yupA**	0.7	0.977	NA	0.709	0.977	NA	0.725	0.955	NA	0.725	0.955	NA	0.673	1	NA	0.66	0.955	NA	0.737	1	NA	0.677	1	NA
**1cjyA**	**1mlaA**	0.563	0.791	NA	0.563	0.791	NA	0.61	0.485	NA	0.64	0.052	NA	0.55	0.097	NA	0.43	0.597	NA	0.633	0.396	NA	0.448	0.761	NA
**1k32A**	**1vaeA**	0.705	0.7	NA	0.756	0.7	NA	0.765	0.533	NA	0.782	0.667	NA	0.514	0.85	NA	0.513	0.783	NA	0.552	0.7	NA	0.518	0.783	NA
**1mvfD**	**1n0gA**	0.753	0.5	2	0.753	0.5	1	0.753	0.5	1	0.753	0.5	1	0.653	1	2	0.651	1	2	0.757	1	2	0.653	1	2
**1e0dA**	**1p3dA**	0.834	0.937	NA	0.842	0.911	NA	0.845	0.937	NA	0.846	0.937	NA	0.819	0.835	NA	0.614	0	NA	0.894	0.848	NA	0.614	0.025	NA
**1m7oA**	**1w0mA**	0.81	0.953	NA	0.849	0.881	NA	0.849	0.881	NA	0.853	0.824	NA	0.79	0.891	NA	0.795	0.979	NA	0.883	0.855	NA	0.793	0.819	NA
**1si7A**	**1z2zA**	0.895	0.909	NA	0.903	0.856	NA	0.904	0.856	NA	0.904	0.856	NA	0.847	0.924	NA	0.76	0.879	NA	0.948	0.89	NA	0.766	0.735	NA
**1a62A**	**1pqhA**	0.434	1	NA	0.57	0	NA	0.57	0	NA	0.57	0	NA	0.282	0	NA	0.26	1	NA	0.534	1	NA	0.282	0	NA
**1d1nA**	**1exmA**	0.743	1	1	0.785	0.37	1	0.785	0.37	NA	0.794	0.891	1	0.681	1	1	0.679	1	1	0.801	1	1	0.685	1	1

		ICARUS L1			ICARUS L2			ICARUS L3			ICARUS L4			FATCAT			DEDAL			KPAX			TMalign		
		TM	Ref.		TM	Ref.		TM	Ref.		TM	Ref.		TM	Ref.		TM	Ref.		TM	Ref.		TM	Ref.	
**1l9gA**	**1oe4A**	0.732	0.932	NA	0.747	0.932	NA	0.767	0.784	NA	0.785	0.705	NA	0.729	1	NA	0.731	1	NA	0.824	0.875	NA	0.732	1	NA
**1j4wA**	**2fmrA**	0.697	0.773	2	0.732	0.636	2	0.754	0.227	2	0.754	0.227	2	0.566	0.636	1	0.619	0.636	1	0.744	0.773	2	0.618	0.636	2
**1bx4A**	**2ajrA**	0.786	0.915	NA	0.795	0.911	NA	0.795	0.911	NA	0.845	0.925	NA	0.753	0.775	NA	0.753	0.878	NA	0.816	0.667	NA	0.755	0.765	NA
**1l9lA**	**1o82A**	0.616	0	NA	0.672	0	NA	0.672	0	NA	0.672	0	NA	0.611	0.537	NA	0.389	0.5	NA	0.796	0.556	NA	0.459	0.185	NA
**1gsoA**	**1w93A**	0.8	0.962	NA	0.808	0.962	NA	0.808	0.962	NA	0.821	0.981	NA	0.763	0.846	NA	0.718	0.962	NA	0.858	0.981	NA	0.729	0.962	NA
**1a49A**	**1o65A**	0.57	0.968	NA	0.616	0.714	NA	0.655	0.635	NA	0.668	0.635	NA	0.519	0.905	NA	0.355	1	NA	0.552	0.952	NA	0.358	0	NA
**1msvA**	**1tluA**	0.764	1	1	0.779	0.794	1	0.804	0.691	1	0.804	0.691	1	0.712	1	1	0.729	1	1	0.824	1	1	0.737	1	1
**1ay9A**	**1b12A**	0.765	0.901	NA	0.769	0.62	NA	0.785	0.634	NA	0.802	0.634	NA	0.657	0.648	NA	0.662	0.944	NA	0.701	0.958	NA	0.675	0.93	NA
**2ackA**	**2cutA**	0.659	0.478	NA	0.701	0.611	NA	0.721	0.456	NA	0.755	0.633	NA	0.565	0.556	NA	0.637	0.856	NA	0.761	0.622	NA	0.64	0.878	NA
**1qo2A**	**1thfD**	0.833	1	1	0.834	1	1	0.841	1	1	0.848	0.722	4	0.822	1	1	0.822	1	1	0.88	1	1	0.824	1	1
**1t3tA**	**1t4aA**	0.808	0.906	1	0.815	0	1	0.825	0	1	0.825	0	1	0.759	0.868	1	0.781	0.962	1	0.882	0.868	1	0.782	0.906	1
**1mr1C**	**1oqjA**	0.663	0.714	NA	0.663	0.714	NA	0.663	0.714	NA	0.663	0.714	NA	0.578	0.911	NA	0.579	0.911	NA	0.661	0.911	NA	0.592	0.911	NA
**1feuD**	**1qtqA**	0.52	0.846	2	0.633	0.846	2	0.745	0.846	2	0.76	0.846	2	0.487	0.846	2	0.307	0.846	2	0.365	0.923	1	0.296	0	1
**1sjgA**	**1vf5D**	0.623	0.561	NA	0.678	0.695	NA	0.678	0.695	NA	0.678	0.695	NA	0.597	0.768	NA	0.552	0.427	NA	0.762	0.585	NA	0.585	0.561	NA
**1kwaA**	**1ujvA**	0.718	1	NA	0.749	1	NA	0.749	1	NA	0.749	1	NA	0.638	1	NA	0.681	0.921	NA	0.777	0.984	NA	0.686	0.921	NA
**1gt8A**	**1o94A**	0.696	0.779	NA	0.71	0.832	NA	0.731	0.768	NA	0.731	0.768	NA	0.499	0.768	NA	0.425	0.853	NA	0.494	0.737	NA	0.427	0.747	NA
**1d5tA**	**1gt8A**	0.366	0	NA	0.466	0	NA	0.517	0	NA	0.517	0	NA	0.555	0.42	NA	0.344	0.42	NA	0.361	0	NA	0.344	0	NA
**1gn0A**	**1jl3A**	0.6	0.96	NA	0.715	0.96	NA	0.759	0.96	NA	0.759	0.96	NA	0.468	0	NA	0.423	0	NA	0.414	0.96	NA	0.428	0	NA
**3ssiA**	**4sgbI**	0.464	0	NA	0.519	0	NA	0.519	0	NA	0.519	0	NA	0.236	0	NA	0.391	0	NA	0.589	0	NA	0.366	0.16	NA
**1ps9A**	**1v0jA**	0.491	0.812	NA	0.542	0.713	NA	0.567	0.65	NA	0.608	0.65	NA	0.463	0.65	NA	0.376	0.963	NA	0.438	0.988	NA	0.377	0.963	NA
**MEAN**		0.686	0.742		0.729	0.662		0.747	0.648		**0.758**	0.635		0.630	0.716		0.587	**0.772**		0.710	0.758		0.598	0.663	

Supplementary Table S16. ICARUS performs two alignments: query vs. target and then target vs. query. For each RIPC target pair we show the absolute difference in terms of TM-score between each exploration level when ICARUS swaps proteins to align.

Protein 1	Protein 2	Level 1	Level 2	Level 3	Level 4
d1an9a1	d1npx_1	0.03	0.025	0.006	0.021
d1ay9b_	d1b12a_	0.055	0.043	0.776	0.782
d1b5ta_	d1k87a2	0.003	0.013	0.027	0
d1crl__	d1ede__	0.042	0.019	0.708	0.008
d1d5fa_	d1nd7a_	0.002	0.003	0.003	0.926
d1dlia1	d1mv8a1	0.003	0.033	0	0
d1gbg__	d1ovwa_	0.005	0.014	0.706	0.069
d1ggga_	d1wdna_	0.002	0.003	0	0.002
d1gsa_1	d2hgsa1	0.023	0.049	0.014	0
d1hava_	d1kxf__	0.004	0.029	0.015	0
d1hcy_2	d1lnlb1	0.104	0.003	0.606	0.648
d1jj7a_	d1lvga_	0.034	0.061	0.617	0.003
d1jwyb_	d1puja_	0.106	0.027	0.011	0.024
d1jwyb_	d1u0la2	0.132	0.027	0.702	0.005
d1kiaa_	d1nw5a_	0.024	0.018	0.047	0.051
d1l5ba_	d1l5ea_	0.01	0.011	0	0
d1nkl__	d1qdma1	0.047	0.777	0	0
d1nls__	d2bqpa_	0.114	0.115	0.021	0.02
d1nw5a_	d2adma_	0.13	0.043	0.03	0.71
d1qasa2	d1rsy__	0.035	0.029	0.762	0
d1qq5a_	d3chy__	0.054	0.048	0.768	0.797
d2adma_	d2hmyb_	0.04	0.056	0.015	0.662

d2bbma_	d4cln__	0.018	0.01	0.002	0
d1adl__	d1mup__	0.006	0.014	0.713	0
d1afra_	d1jkua_	0.046	0.044	0.705	0.704
d1aj3__	d2spca_	0.151	0.064	0.003	0
d1aqza_	d1a2pa_	0.027	0.018	0	0
d1b09a_	d1dy4a_	0.027	0.012	0.635	0.069
d1b6a_1	d1bia_1	0.153	0.675	0	0
d1dy4a_	d2sak__	0.056	0.084	0	0.694
d1cpo_1	d1cpo_2	0.151	0.005	0.629	0
d1d5ra1	d1rsy__	0.067	0.078	0.682	0
d1dmaa_	d1lt3a_	0.005	0.002	0.057	0.62
d1ed9a_	d1p49a_	0.029	0.066	0.57	0.573
d1he9a_	d1nfn__	0.016	0.005	0.051	0.795
d1hx6a1	d1p2za2	0.017	0.025	0.004	0.607
d1lzy__	d148le_	0.064	0.043	0.013	0.019
d1ng4a2	d3cox_2	0.015	0.025	0.715	0
d1olza2	d2trcb_	0.031	0.052	0.064	0.004
d1xyza_	d2hvm__	0.03	0.027	0.022	0.714
	**MEAN**	0.048	0.067	0.267	0.238
